# Unveiling the Potential of Ambient Air Annealing for Highly Efficient Inorganic CsPbI_3_ Perovskite Solar Cells

**DOI:** 10.1021/jacs.3c11711

**Published:** 2024-02-09

**Authors:** Zafar Iqbal, Roberto Félix, Artem Musiienko, Jarla Thiesbrummel, Hans Köbler, Emilio Gutierrez-Partida, Thomas W. Gries, Elif Hüsam, Ahmed Saleh, Regan G. Wilks, Jiahuan Zhang, Martin Stolterfoht, Dieter Neher, Steve Albrecht, Marcus Bär, Antonio Abate, Qiong Wang

**Affiliations:** † Helmholtz-Zentrum Berlin für Materialien und Energie GmbH, Hahn-Meitner-Platz 1, 14109 Berlin, Germany; ‡ Institute for Physics and Astronomy, University of Potsdam, Karl-Liebknecht-Straße 24−25, 14476 Potsdam-Golm, Germany; § Electronic Engineering Department, The Chinese University of Hong Kong, Hong Kong 999077, SAR China; ∥ Department of Chemistry and Pharmacy, Friedrich-Alexander-Universität Erlangen-Nürnberg (FAU), Egerland Street 3, 91058 Erlangen, Germany; ⊥ Helmholtz Institute Erlangen-Nürnberg for Renewable Energy (HI ERN), Albert-Einstein-Street 15, 12489 Berlin, Germany; # Department of Chemistry, Bielefeld University, Universitätsstraße 25, 33615 Bielefeld, Germany; ∇ Clarendon Laboratory, University of Oxford, Parks Road, Oxford OX1 3PU, UK; ○ Energy Materials In-situ Laboratory Berlin (EMIL), Helmholtz-Zentrum Berlin für Materialien und Energie GmbH, 12489 Berlin, Germany

## Abstract

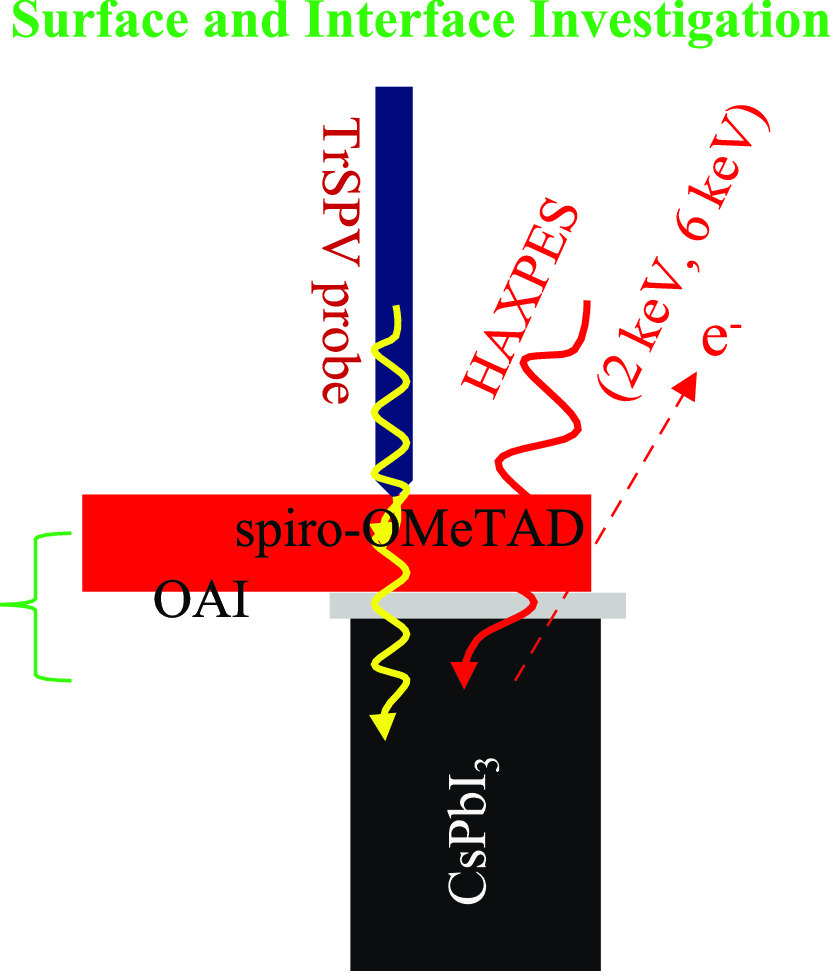

Here, we report a detailed surface analysis of dry- and ambient air-annealed CsPbI_3_ films and their subsequent modified interfaces in perovskite solar cells. We revealed that annealing in ambient air does not adversely affect the optoelectronic properties of the semiconducting film; instead, ambient air-annealed samples undergo a surface modification, causing an enhancement of band bending, as determined by hard X-ray photoelectron spectroscopy measurements. We observe interface charge carrier dynamics changes, improving the charge carrier extraction in CsPbI_3_ perovskite solar cells. Optical spectroscopic measurements show that trap state density is decreased due to ambient air annealing. As a result, air-annealed CsPbI_3_-based *n*–*i–p* structure devices achieved a 19.8% power conversion efficiency with a 1.23 V open circuit voltage.

## 1 Introduction

Metal halide perovskites (MHPs) have emerged as promising candidates in the optoelectronic industry owing to their unique optoelectronic properties such as tunable bandgap,^1−3^ long charge carrier diffusion length,^4,5^ high defect tolerance,^6,7^ and high light absorption coefficient.^8,9^ Since Miyasaka et al. in 2009 first reported the use of halide perovskites as light absorbers in solar cells reaching power conversion efficiencies (PCE) of 3.8%,^10^ the PCE of corresponding devices has climbed to 26.1% for single junction solar cells today,^11^ surpassing the record PCE set by contemporary photovoltaic thin-film technologies based on e.g., copper indium gallium selenide (CIGS) (23.4%), CdTe (22.1%), and organic (19.2%) light absorbers,^12,13^ thus now approaching the detailed balance limit (∼33%).^14−16^ However, the long-term stability of halide perovskite solar cells (PSC) lags significantly because of the presence of volatile organic cations, such as methylammonium (MA^+^), in the crystal structure and its sensitivity to high temperature and moisture.^17−19^ Replacing the volatile organic cations with Cs^+^, i.e., the development of inorganic halide perovskites expressed as CsPbX_3_ (where X stands for halide, such as chloride, bromide, and iodide) is hence one mitigation strategy that is studied in the research field.^20^ Inorganic halide perovskites exhibit high thermal stability at temperatures exceeding 300 °C.^21^ Among the CsPbX_3_ compounds, CsPbI_3_ has the lowest optical bandgap, *E*_g_ (∼1.7 eV),^22^ making it an excellent light absorber candidate for the top cell in a tandem structure with silicon or a narrow *E*_g_ perovskite as the bottom cell light absorber.^23^ Besides, inorganic halide perovskites promise to outperform their organic–inorganic hybrid counterparts in terms of long-term stability at elevated operating temperatures (reaching 85 °C).^24^ For these reasons, CsPbI_3_ has received significant attention in the perovskite photovoltaic community.^25^

CsPbI_3_ undergoes crystal phase transitions, transforming from a cubic (α) phase to a tetragonal (β) phase, to an orthorhombic (γ) phase as its temperature decreases from above 300 °C to room temperature.^26^ Due to the low tolerance factor in CsPbI_3_, maintaining phase stability at room temperature requires special attention.^27^ Furthermore, obtaining pinhole-free CsPbI_3_ films with low defect densities remained challenging.^28,29^ However, controlling the nucleation and crystallization of CsPbI_3_ has been suggested to overcome this obstacle, with many efforts made to this end.^30−34^ In addressing this challenge, Wang et al. introduced dimethylammonium iodide (DMAI) in the precursor of CsPbI_3_, which resulted in a stabilized β-phase with an *E*_g_ of 1.68 eV.^35^ Further, the postpassivation with long-chain organic halides, such as choline iodide (CHI) and large cation halide, phenyltrimethylammonium chloride (PTACl), increases the PCE of resulting devices up to 19%.^35,36^ The role of DMAI, whether it remains in the final crystal structure of CsPbI_3,_ has been investigated.^21,29,36^ For example, it has been reported that DMAI sublimes in dry air while controlling the crystallization kinetics of CsPbI_3_ films.^36^ Generally, the absorber for perovskite solar cells is annealed in the glovebox (with a controlled environment) or dry air box (with oxygen contents). These controlled environments significantly increase the operational costs for industrial processes, so ambient environment annealing will be cost-effective.^37^ The necessity of annealing in a dry airbox instead of a nitrogen-filled glovebox was also investigated.^38,39^ It is found that annealing in the presence of oxygen removes the organic cations in the precursor solution and passivates halide vacancies, resulting in a higher open circuit voltage (*V*_OC_).^40^

For all-inorganic perovskite-based devices, the open circuit voltage deficit (*V*_loss_) is higher than that in all corresponding compositions of organic–inorganic halide perovskite solar cells.^41,42^ Thus, the record *V*_OC_ of devices based on CsPbI_3_ is still limited to ∼1.25 V.^43−45^ The *V*_OC_ of the solar cells depends on the interfacial physical properties.^46−51^ Until today, there has existed an information gap for CsPbI_3_-based solar cells, such as the effect of the annealing environment on the surface chemistry, its subsequent interface, and charge extraction dynamics, which have been suggested to be the main *V*_OC_ bottlenecks.^52^ We have used a state-of-the-art method for CsPbI_3_ film preparation and device fabrication^53^ in our study to address the mentioned knowledge gap.

In this work, we report a detailed analysis of the interface between CsPbI_3_ and the hole transport material (HTM) and how its properties are affected by annealing in ambient air and a dry air box. We conducted steady-state photoluminescence spectroscopy (stPL) and time-resolved photoluminescence spectroscopy (TRPL) to determine the emission and charge carrier lifetimes. In addition, transient surface photovoltage (trSPV) measurements were conducted to investigate charge carrier extraction at the interface. At the same time, hard X-ray photoelectron spectroscopy (HAXPES) was used to probe the chemical and electronic structure of the differently annealed CsPbI_3_ and perovskite/hole transport layer interface. Finally, we fabricated CsPbI_3_-based devices. Using ambient air-annealed absorbers, PCE values of up to 19.8% have been reached (representing an improvement of >1% on an absolute scale compared to cells based on dry air-annealed absorbers). This improvement is mainly due to a systematic *V*_OC_ gain, reaching values up to 1.23 V, demonstrating that the annealing of CsPbI_3_ films in ambient air is economically beneficial. Hence, this work not only paves the way for new fabrication strategies involving ambient air annealing processing but also contributes to solving the main challenge of *V*_OC_ deficit (*V*_loss_) with a reduction of ∼ 0.47 V.

## 2 Results and Discussion

### 2.1 Film Characterization

In recent work on CsPbI_3_, researchers are using DMAI as a precursor along with PbI_2_ and CsI. This organic molecule (DMAI) sublimes at ∼90 °C during annealing in dry air-box conditions with controlled humidity.^36^ The annealing step/environment plays a vital role in the crystallization and growth and, thus, the overall quality of the absorber material.^54−57^ Here, we report the impact of two different annealing processes on the properties of CsPbI_3_: One approach is based on annealing in a dry air box with relative humidity (RH) controlled to be at ∼1% (hereafter, referred to as “dry air”), and the other process takes place in ambient air with RH between ∼45 to 50% (hereafter, referred to as “ambient air”). These absorbers were subsequently capped with a 10–20 nm layer of *n*-octylammonium iodide (*n*-OAI) with a 2D structure, improving their stability.^53,58^ As a reference, we also study dry air-annealed CsPbI_3_ absorbers without *n*-OAI (hereafter, referred to as “OAI-free”).

**Figure 1:**
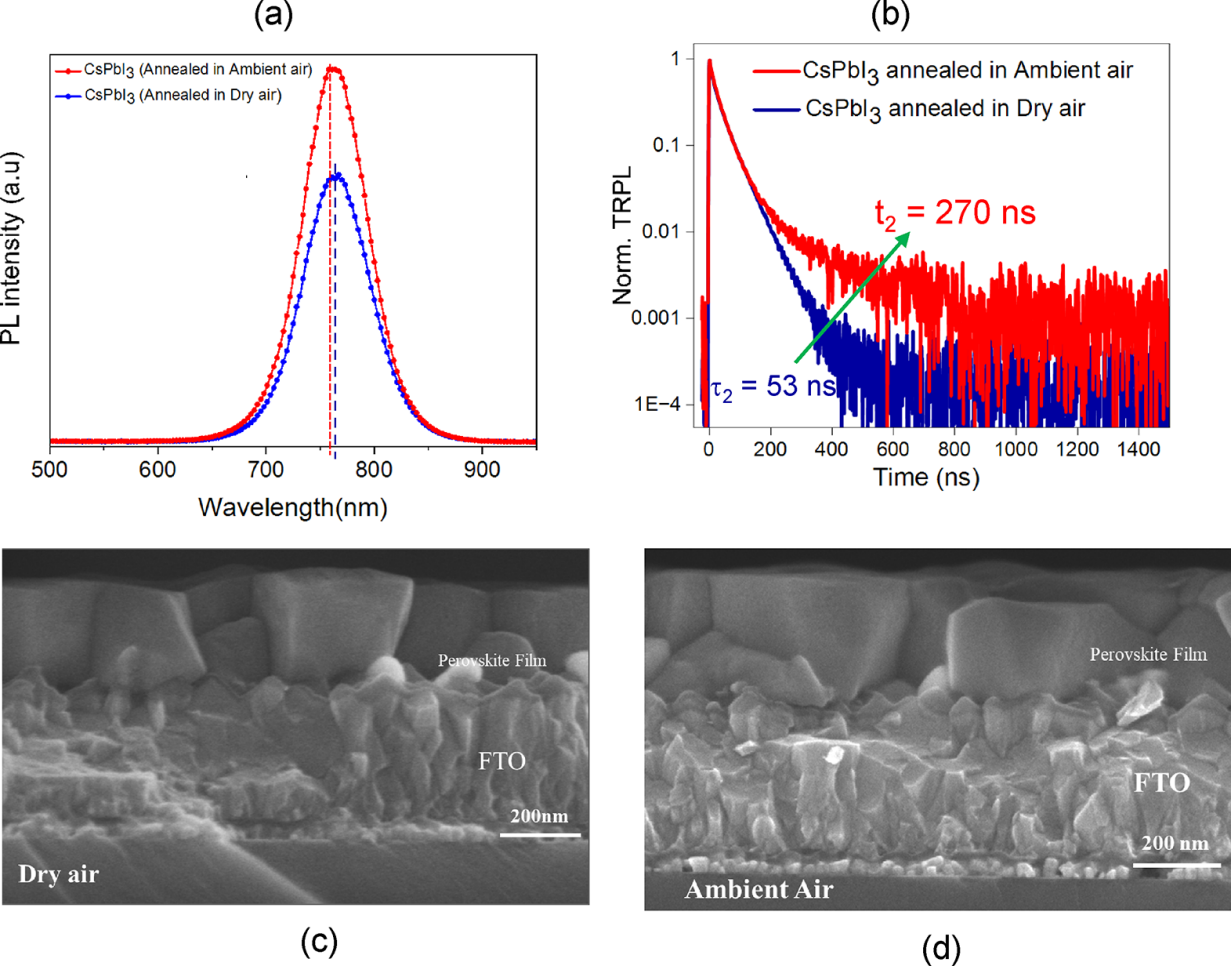
(a) Steady-state photoluminescence emission spectra of dry air and ambient air annealed CsPbI_3_ absorbers. (b) Time-resolved photoluminescence (TRPL) decay of dry air and ambient air annealed CsPbI_3_ films (fitted spectra are shown in Figure S4a,b). Cross-sectional SEM images of dry air (c) and ambient air, (d) annealed CsPbI_3_ absorber films were deposited on FTO/TiO_2_–C substrates.

Initial characterization of the absorbers with ultraviolet–visible (UV–vis) absorption spectroscopy (Figure S1) shows that both dry air and ambient air-annealed CsPbI_3_ films have an absorption edge at around 735 nm, indicating *E*_g_ values of ∼1.69 eV (see Tauc plots of UV–vis spectra in Figure S2), which is inconsistent with the *E*_g_ values (1.69–1.71 eV) for CsPbI_3_ available in the literature.^36,42,53^ Steady-state photoluminescence (stPL) emission spectroscopy was conducted using an excitation light with a wavelength of 445 nm on the top side of the film (perovskite side). The excitation wavelength and illumination direction are important in PL analysis to know the penetrations and surface passivation.^59^ stPL reveals a PL peak emission at 764 nm for the dry air-annealed CsPbI_3_, while for the ambient air-annealed CsPbI_3_, the PL peak is slightly blue-shifted to 762 nm (see Figure 1a). The higher PL intensity and blue shift in the excitation peak indicate defect passivation on the surface of the ambient air-annealed CsPbI_3_ film.^39,59−61^ TRPL spectra measured on Glass/TiO_2_/CsPbI_3_: OAI dry air and ambient air annealed samples, given in Figure 1b, were fitted with the biexponential eq S8. The fitted parameters for the TRPL spectra are presented in Figure S3a,b and values in Table S1. We attribute the initial exponential decay (*t*_1_) to charge transfer to TiO_2_ and the second exponential decay (*t*_2_) to nonradiative interfacial recombination.^33,62^ Overall, TRPL is consistent with the stPL measurements and confirms that air-annealed samples have comparatively fewer defects.^33,35,36,60^ Furthermore, we analyzed the morphology of the annealed CsPbI_3_ films. Figure 1c,d shows the cross-sectional scanning electron microscopy (SEM) images for the dry air and ambient air annealed absorbers, which show both films have similar thicknesses (∼350 nm). Top-view SEM images confirm this (see Supporting Information, SI, Figure S4a,b), which show that the CsPbI_3_ films exhibit similar compact, smooth, and pinhole-free morphologies, with similar grain sizes, i.e., ∼500 nm. The SEM images also show the absence of “fuzzy” grains previously attributed to remainders of unreacted precursors (e.g., DMAI).^36^

### 2.2 Surface Chemistry and Electronic Structure Profile Investigation by HAXPES

**Figure 2:**
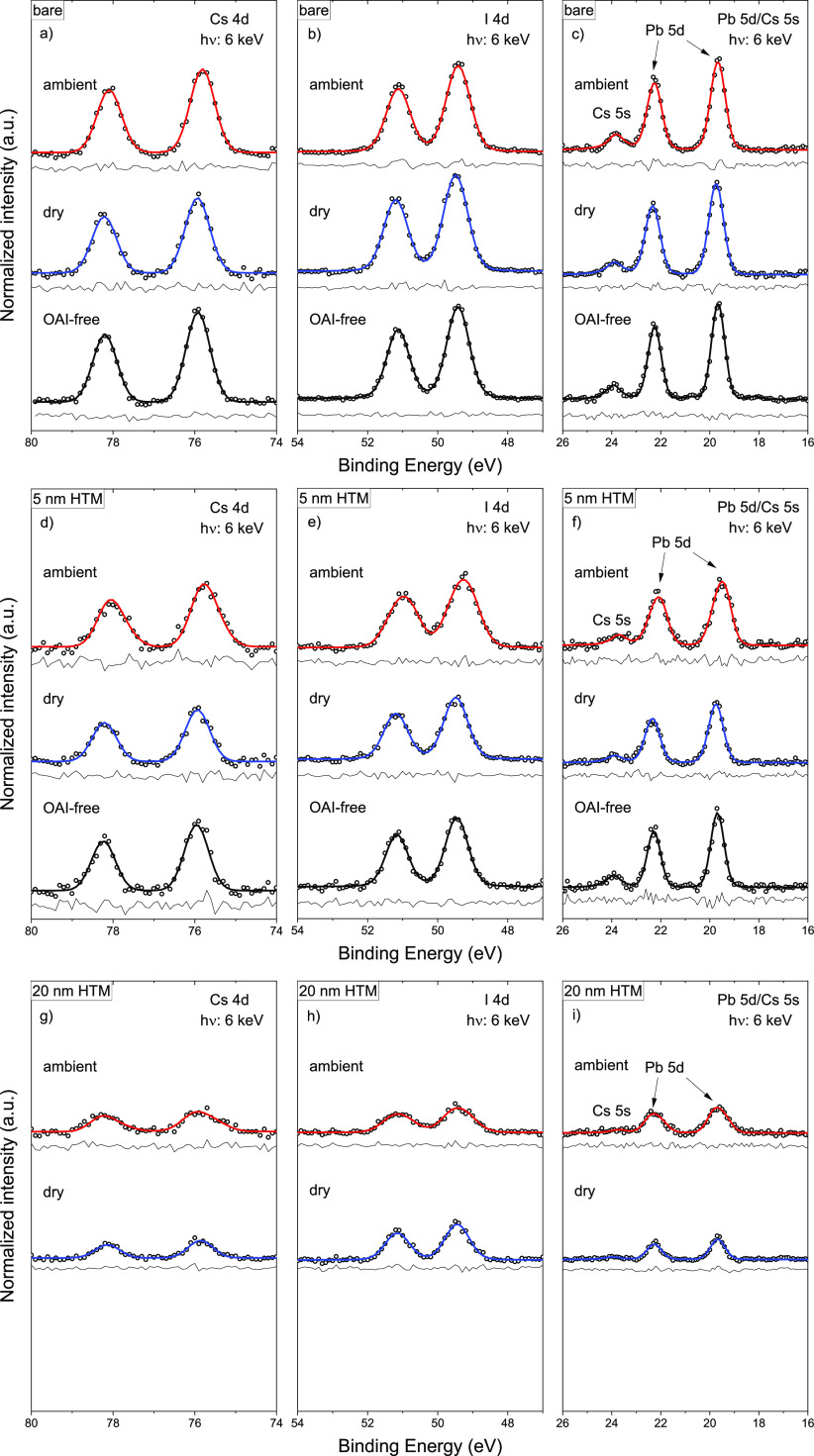
HAXPES detail spectra of the Cs 4d (a, d, g), I 4d (b, e, h), and overlapping Pb 5d/Cs 5s (c, f, i) photoemission lines for the variously treated CsPbI_3_ films (i.e., OAI-free, dry air, and ambient air annealed) with 0 nm (i.e., bare) (a, b, c), 5 nm (d, e, f), and 20 nm (g, h, i) films of spiro-OMeTAD, respectively. The spectra were measured using 6 keV excitation and normalized to background intensity, with vertical offsets added for clarity. Curve fit results are included.

The surface and interface chemical modifications induced by two different annealing atmospheres were characterized by using HAXPES. For this purpose, the CsPbI_3_ samples were deposited on F-doped tin oxide (FTO) coated glass substrates, a transparent conducting oxide material used in solar cell devices. Besides the bare CsPbI_3_ absorber samples, we have made a series of additional HTM/CsPbI_3_ samples also to study the interface properties, i.e., on the dry air annealed with an OAI-free as well as on the dry air and ambient air annealed CsPbI_3_ absorbers with *n*-OAI passivation, spiro-OMeTAD based HTM layers of different (nominal) thicknesses of 0 nm (bare), 5, 20, and 200 nm were deposited. HAXPES survey spectra of the bare samples in Figure S5a confirm the presence of all expected elements: Cs, Pb, and I-related photoemission signals were observed in all samples, and C and N-related photoemission signals were observed only in the OAI-treated samples. Contrarily to previous reports that terminal ions make bonds with oxygen at the surface,^39^ no visible contamination by e.g., oxygen is seen to result from the annealing treatments in different RH, and close examination of the most prominent halide perovskite-related HAXPES peaks, Cs 3d, I 3d, and Pb 4f (Figures S6 and S7) and the corresponding shallow core level peaks, Cs 4d, I 4d, and Pb 5d (Figures 2 and S8) show no significant change in line shape between the measurements of the dry air and ambient air annealed (and *n*-OAI passivated) CsPbI_3_ absorbers. However, the corresponding photoemission lines of the OAI-free CsPbI_3_ seem to be systematically narrower, which is attributed to the formation of an *n*-OAI-modified thin surface layer (with slightly different chemical and electronic structure compared to that of the OAI-free CsPbI_3_ absorber). Furthermore, the OAI-free CsPbI_3_ absorber also exhibits a small signal from metallic Pb, which is absent in the *n*-OAI treated samples, an effect most easily detected in Figures S6c and S8c. The survey spectra of the HTM thickness series in Figure S5b–d are consistent with the growth of a spiro-OMeTAD-based HTM (now also exhibiting F and S-related HAXPES contributions from LiTSFI and FK 209, which are included in the HTM-mixture, see discussion in SI), which increasingly attenuates the photoemission signal from the underlying CsPbI_3_ as it becomes thicker, with only HTM-related signals appearing for the sample with a 200 nm thick HTM. There is little or no difference in the growth and composition of the HTM layer on the differently prepared CsPbI_3_ films, except for the 5 nm data set, where a clear F 1s signal is present only in the spectrum measured on the sample prepared with an OAI-free CsPbI_3_. Quantification of the Cs:Pb:I stoichiometry for the 0, 5, and 20 nm (shown in Figure S9a,b based on the measurements of perovskite-related shallow core levels using 2 and 6 keV, respectively; for more details, see the experimental section in the SI) confirms that the elemental composition is unchanged as a result of either the different annealing processes or by the deposition of the HTM, with the most significant differences being the slightly elevated I and decreased Pb photoemission line intensities associated with the presence of the OAI-modified surface layer. This stability of the CsPbI_3_ composition and its good agreement with the nominal composition suggest that no extensive chemical reactions have occurred at the surface of the bare CsPbI_3_ absorbers or the corresponding interfaces to the HTM.

**Figure 3:**
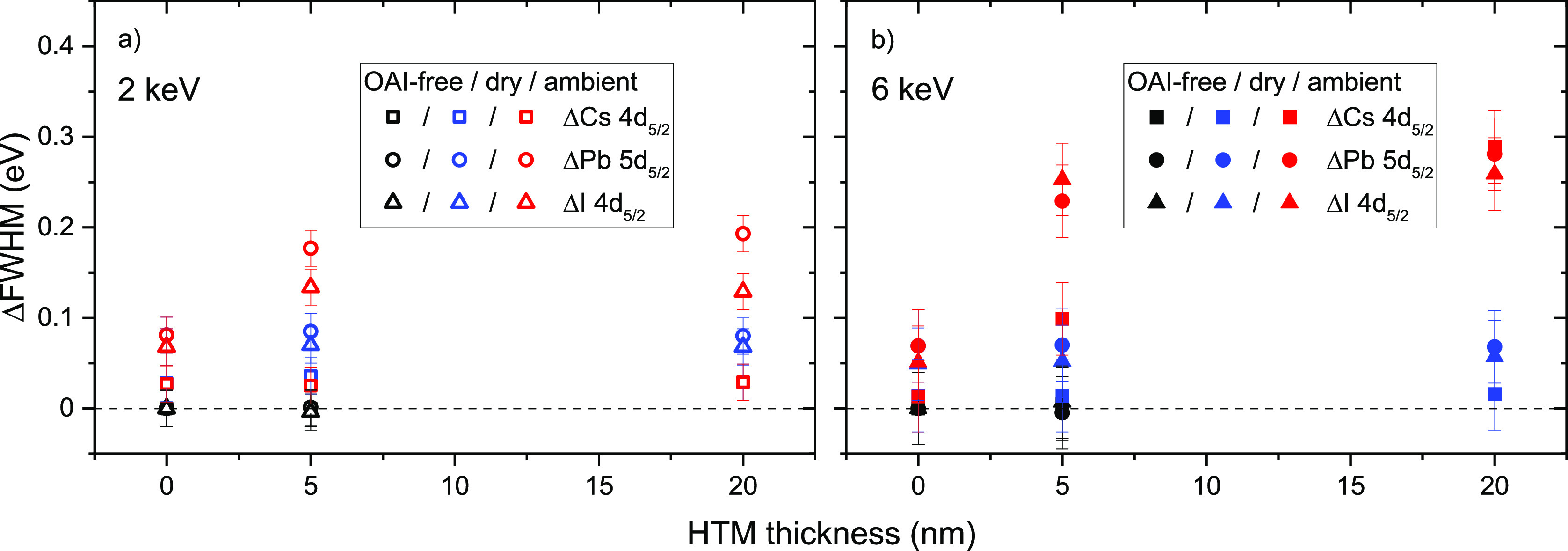
Changes in full-width-half-maximum (FHWM) values of the HAXPES Cs 4d_5/2_, Pb 5d_5/2_, I 4d_5/2_ peaks of CsPbI_3_ absorbers annealed in dry or ambient air with 0 nm (i.e., bare), 5 and 20 nm films of HTM, measured with excitation energies of (a) 2 and (b) 6 keV (derived from the spectra shown in Figures S8 and 2), compared to the corresponding peak FHWM values of the bare, OAI-free CsPbI_3_ sample.

Despite this apparent chemical stability, close inspection of the collected HAXPES HTM/CsPbI_3_ data reveals that there is a slight change in the line widths of the perovskite-derived core level spectra between the dry air and ambient air annealed CsPbI_3_ absorbers: the photoemission lines collected for the samples based on CsPbI_3_ absorbers annealed in ambient air are slightly broader in all cases, as presented in Figure 3a,b. Such a broadening can arise from changes in chemical structure (i.e., formation of new species beneath the HTM) or electronic structure profiles. Due to the relatively high information depth of HAXPES measurements, probing a sample volume in which band bending occurs may result in a line broadening^63−66^ rather than or in addition to a shift in peak position. As discussed above, we do not see any indications of pronounced chemical changes in the CsPbI_3_ films. However, because of the same high HAXPES information depth-induced integration effect, the formation of a thin interlayer (similar to the differences we observe due to the occurrence of OAI-induced surface modification) cannot be ruled out. In the case that one would attribute the observed line broadening to be due to a band bending enhancement, one could speculate that the ambient air annealing alters the defect distribution and concentration in the CsPbI_3_ absorber, allowing for a more pronounced upward band bending in the absorber upon interface formation with the HTM. An interface-formation-induced upward band bending in the absorber, i.e., a binding energy (BE) shift of the absorber-related photoemission lines toward the Fermi-level (reducing the binding energy) can indeed be observed when comparing the halide perovskite-related photoemission lines for the HTM/CsPbI_3_ samples based on absorbers annealed in ambient and dry air. The BE of the Cs 3d, I 3d, and Pb 4f (Figures S6 and S7) lines and of the corresponding shallow core level peaks Cs 4d, I 4d, and Pb 5d (Figures 2 and S8) of the HTM/CsPbI_3_ samples based on the absorber annealed in ambient air are systematically lower than those made from absorbers annealed in dry air. (Shifts in BE of the peaks Cs 4d_5/2_, I 4d_5/2_, and Pb 5d_5/2_ peaks of the investigated samples compared to BE values of the bare, OAI-free CsPbI_3_ sample are presented in Figure S9c,d.) This effect is most pronounced for the samples with a nominal 5 nm thick HTM layer, while we do not see for the HTM/CsPbI_3_ samples with a nominal 20 nm Spiro-OMeTAD film.

### 2.3 Charge Carrier Extraction Dynamics and Trap Passivation

**Figure 4:**
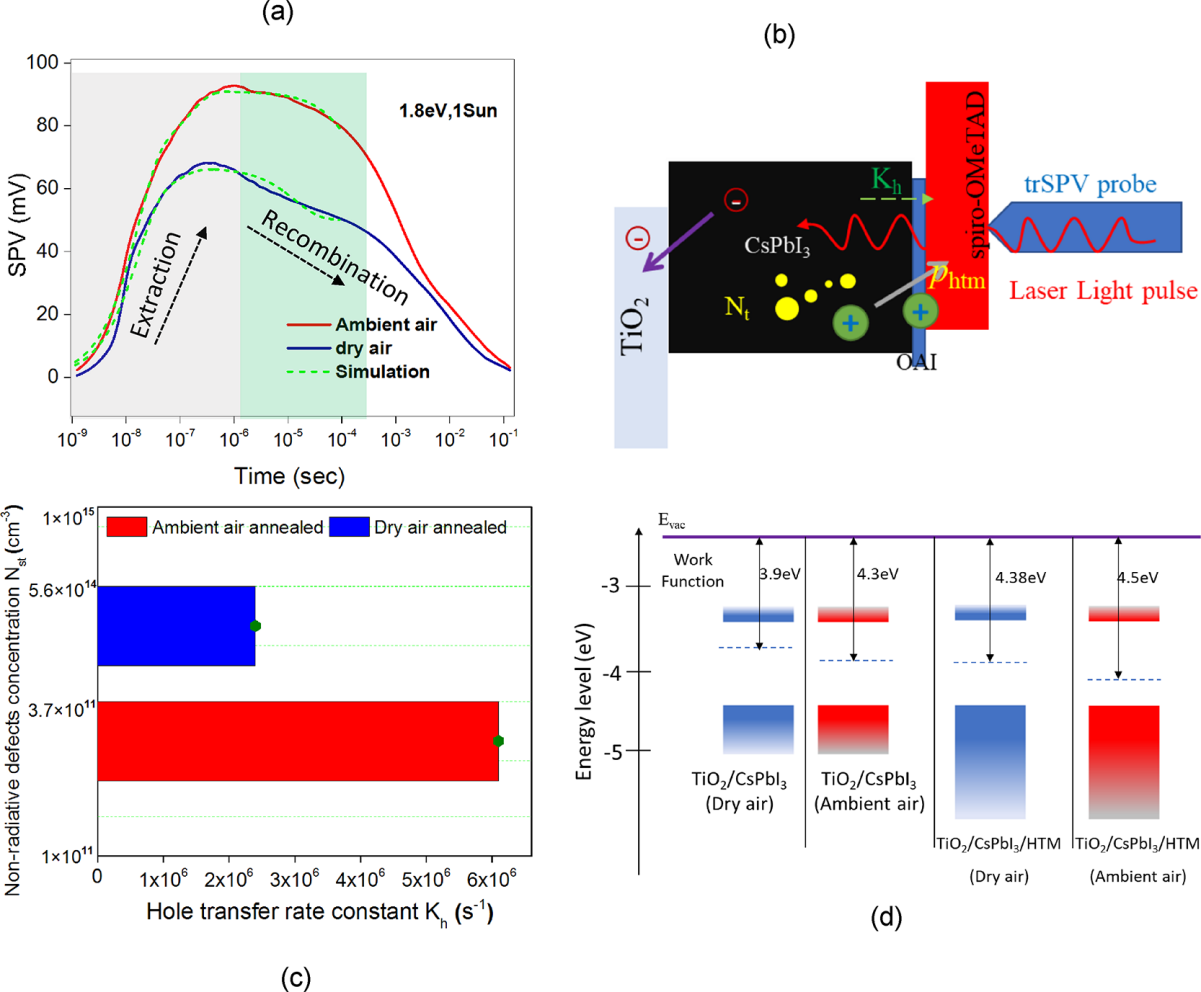
Interfacial dynamics. (a) Surface photovoltage (SPV) measurements for ETM/CsPbI_3_/HTM samples based on absorbers annealed in dry and ambient air. The samples were measured under 1 sun illumination and using 1.8 eV laser excitation and recorded in ambient air conditions. The extracted hole concentrations *p*_htm_ induce the simulated trSPV signal, shown by green curves. (b) Charge extraction and recombination model describing carrier transport to the HTM layer. The constant *K*_h_ corresponds to hole injection rates from the perovskite to the HTM side. Defect concentration *N*_t_ is responsible for Shockley-Read-Hall recombination (SRH) of nonradiative recombination. (c) *K*_h_ and nonradiative defect concentration *N*_st_ values were extracted from the fitting. (d) Work function measurements with the Kelvin Probe method for dry air-annealed and ambient air-annealed samples.

We further investigated the change in interface dynamics induced by the annealing medium. Charge carrier extraction by hole transport layer (HTL) and electron transport layer (ETL) is essential to PSC. The hole extraction rate coefficient (*K*_h_) determines how fast holes can be extracted from the active perovskite absorber to the selective HTL layer. The knowledge of *K*_h_ for different HTL interfaces allows them to cross-compare their hole extraction capabilities.^62^ The fast extraction—1/*K*_h_ < carrier lifetime—ensures the collection of the carriers before their recombination in the active material or surface.^67,68^ Further, transient surface photovoltage (trSPV) measurements were conducted to study charge carrier extraction dynamics for two devices (FTO/TiO_2_/Perovskite: OAI/spiro-OMeTAD) annealed at different conditions (i.e., dry air and ambient air). To generate charge carriers, we used a 5 ns light pulse with a photon energy of 1.8 eV and fluence equal to 1 sun (more details in SI). The spectral dependence trSPV mapping is shown in Figure S11. The trSPV measurements revealed a significant boost in the amplitude and rise speed of the ambient air-annealed sample signal compared to the dry air-annealed sample, as shown in Figure 4a. The increase in the trSPV signal can be caused by more efficient charge extraction, larger *K*_h_ or suppressed trap concentration.^42,62,67,68^ Another piece of evidence on more favorable band bending at the surface of the ambient air facilitating hole extraction comes from the Kelvin probe measurements as shown in Figure 4d, detailed in Figure S12, values added in Table S2. It is found that the work function increases from 3.88 eV for the dry air to 4.30 eV for the ambient air annealed sample, which is now much closer to that of the HTM layer. The change in work function (WF) is in line with the upward band bending inferred from the HAXPES data. Both trap passivation and favorable band bending can enhance the charge extraction at the HTL interface. To resolve two phenomena, we have applied charge carrier simulation based on the kinetic equation shown in our previous work^42^ to access the charge carrier extraction rate constant and concentration of traps at the perovskite surface (Details and results of the simulation can be found in the SI and Tables S3 and S4.) After fitting experimental results, we found that the ambient air annealed sample has a three times faster charge carrier extraction rate, which resulted in the more rapid rise of the trSPV signal, as shown in Figure 4c. It shows that the air-annealed sample also exhibits a much lower concentration of defects (5.6 × 10^11^ cm^–3^ compared to 3.7 × 10^14^ cm^–3^ as derived for the dry air-annealed selection) at the perovskite surface, which may act as charge carrier recombination centers. The results suggest that air annealing enhances hole extraction and at the same time passivates defects, thereby improving charge carrier collection at the HTL interface.

### 2.4 Device Performance

We have used dry and ambient air annealed halide perovskite CsPbI_3_ absorbers to fabricate **n*–*i–p** devices with the structure: FTO/compact-TiO_2_/CsPbI_3_/OAI/spiro-OMeTAD/Au. After fabrication of the device, the device was stored in a dry air box to oxidize the spiro-OMeTAD layer. This oxidation induces changes in the lowest unoccupied molecular orbital (LUMO) levels of spiro-OMeTAD, making it more *p*-type.^69,70^ However, the O_2_-soaking duration required for reaching the highest device performance for ambient air and dry air annealed light absorbers differs, as shown in Figure S13. It shows that devices based on halide perovskite absorbers annealed in dry air need longer oxygen soaking than cells based on CsPbI_3_ annealed in ambient air. The underlying reason for this difference could be related to the enhanced band bending suggested for the samples annealed in ambient air, presumably establishing proper interface energetics without tuning the doping concentration of the HTM.

**Figure 5:**
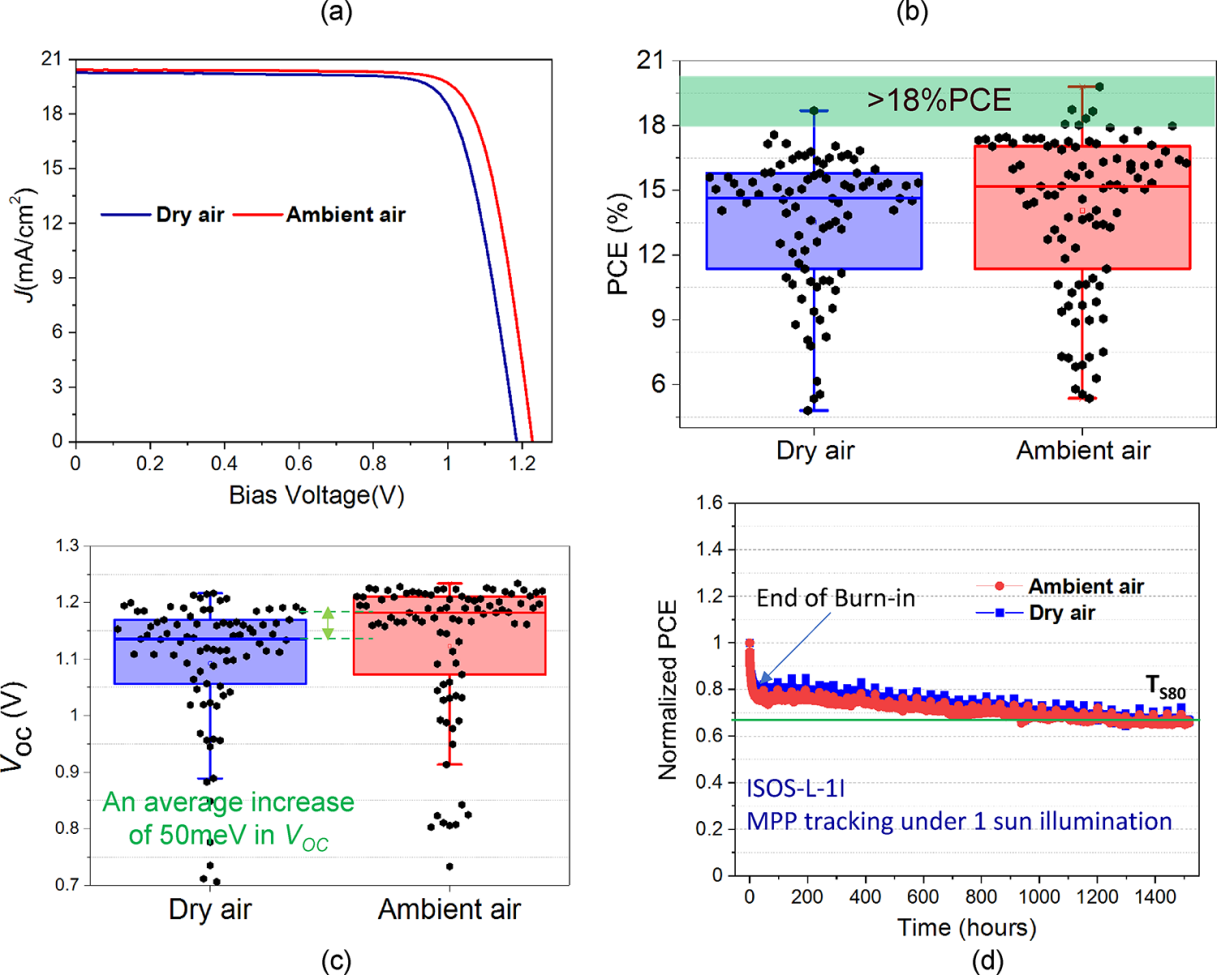
(a) *J–V* curve for champion dry air and ambient air annealed CsPbI_3_ film-based devices. Box chart statistics of 18 dry air and 18 ambient air annealed film-based individual devices with a total of over 90 pixels showing corresponding. (b) PCE and (c) *V*_OC_ values. (d) Long-term stability measurements for 1500 h for dry and ambient air-annealed CsPbI_3_ film-based devices.

Figure 5a shows *J–V* curves for the champion devices. The ambient-air annealed absorber device exhibits a PCE of 19.8%, a *V*_OC_ of 1.23 V, a current density (*J*_SC_) of 20.4 mA/cm^2,^ and a fill factor (*FF*) of 78.9%, clearly outperforming the champion dry-air annealed device, reaching a PCE of 18.6%, a *V*_OC_ of 1.18 V, a *J*_SC_ of 20.2 mA/cm^2^ and an *FF* of 77.76% (Table S5 and Figure S14). We have made 36 devices (18 for each absorber annealing condition), each containing 6 pixels (individual cells). The performance statistics are summarized in Figures 5b,c, and S15 and S16, corroborating a clear statistical difference between the cells based on differently annealed CsPbI_3_ absorbers. For example, there is almost a 50 meV increase in *V*_OC_ for the devices based on the ambient-air annealed absorbers. Figure S17 shows the external quantum efficiency (EQE) measurements for the champion dry- and ambient-air annealed absorber devices. It shows an increase in EQE for the solar cell based on the ambient-air annealed CsPbI_3_ in the wavelength range of 619–710 nm, which is attributed to a reduction in nonradiative recombination.^71^ The *J*_SC_ calculated from EQE is 20.39 and 20.42 mA/cm^2^ for devices based on dry-air and ambient-air annealed CsPbI_3_, respectively. Finally, the long-term stability of both types of devices was measured for 1500 h. We have performed measurements of the devices at maximum power point (MPP tracking) under 1.2 sun illumination following ISOS-L-1I protocol.^72^ Four devices for each condition were measured. Figure 5d shows the normalized PCE over time for 13,13 pixels for dry and ambient air-annealed sample devices. We can explain their stability in terms of *T*_S80_. At the same time, *T*_s80_ is when there is a 20% drop in the PCE of the device after burn-in.^69,73^ Both devices show similar stability with burn-in in the initial few hours dedicated to ion migration^74−76^ and then similar *T*_S80_ values.

## 3 Conclusions

We have conducted a detailed surface and interfacial study for CsPbI_3_ in perovskite solar cells and how they are impacted by the postpreparation annealing step in a dry air box and in an ambient air environment. We have found that the CsPbI_3_ film annealed in ambient air has fewer defects, confirmed by PL and TRPL. A detailed HAXPES study of the surface and interface properties suggests an ambient-air-annealing-induced band bending enhancement at the CsPbI_3_/HTM interface, presumably caused by the passivation of defect states. However, it confirms no chemical changes on the CsPbI_3_ surface while annealing. By trSPV, we see improving charge carrier extraction and low nonradiative interfacial defects for air-annealed samples due to trap passivation and favorable surface band bending. The perovskite solar cells based on ambient air-annealed CsPbI_3_ result in a PCE of 19.8%, a *V*_OC_ of 1.23 V, and *T*_s80_ up to 1500 h. This overall study opens the horizons for new annealing strategies and interfacial studies focusing on inorganic perovskite solar cells as a tool to reduce *V*_loss_.

## Supporting Information Available

Detailed experimental method and device fabrication method; thin film characterization, UV–vis spectroscopy, SEM analysis, HAXPES survey spectra, and detailed analysis of the measurements; contour plots of the transient SPV, transient SPV fitting results, and simulation details; Kelvin probe measurements; champion *J–V* of CsPbI_3_ solar cells and EQE; and box charts of PCE, *J*_sc_, *V*_oc_, and *FF* (PDF)
