# Supplementary material for: Unveiling the Potential of Ambient Air Annealing for Highly Efficient Inorganic CsPbI3 Perovskite Solar Cells
Source: J Am Chem Soc. 2024 Feb 9;146(7):4642–51. doi: 10.1021/jacs.3c11711 (PMC10885157; doi:10.1021/jacs.3c11711)
Supplement: Supplementary file 1 — ja3c11711_si_001.pdf [file ja3c11711_si_001.pdf]

**SUPPLEMENTAL INFORMATION**

# Unveiling the Potential of Ambient Air Annealing for Highly-Efficient Inorganic $CsPbI_3$ Perovskite Solar Cells

Zafar Iqbal[1], Roberto Félix[1], Artem Musiienko[1], Jarla Thiesbrummel[2,3], Hans Köbler[1], Emilio Gutierrez Partida[2], Thomas W. Gries[1], Elif Hüsam[1], Ahmed Saleh[1], Regan G. Wilks,[1,4] Jiahuan Zhang [1], Martin Stolterfoht [2,6], Dieter Neher [2], Steve Albrecht[1], Marcus Bär[1,4,7,8], Antonio Abate[1,9*], Qiong Wang[1*]

[1]Helmholtz-Zentrum Berlin für Materialien und Energie GmbH. Hahn-Meitner-Platz 1, 14109 Berlin, Germany.

[2]Institute for Physics and Astronomy, University of Potsdam, Karl-Liebknecht-Straße 24−25, 14476 Potsdam-Golm, Germany.

[3]Clarendon Laboratory, University of Oxford, Parks Road, Oxford, OX1 3PU UK.

[4]Energy Materials In-situ Laboratory Berlin (EMIL), Helmholtz-Zentrum Berlin für Materialien und Energie GmbH, 12489 Berlin, Germany.

[6]The Chinese University of Hong Kong, Electronic Engineering Department, Hong Kong 999077, SAR China.

[7]Department of Chemistry and Pharmacy, Friedrich-Alexander-Universität Erlangen-Nürnberg (FAU), Egerland Str. 3, 91058 Erlangen, Germany.

[8]Helmholtz Institute Erlangen-Nürnberg for Renewable Energy (HI ERN), Albert-Einstein-Str. 15, 12489 Berlin, Germany.

[9]Department of Chemistry Bielefeld University, Universitätsstraße 25, 33615 Bielefeld, Germany

Corresponding Authors*

antonio.abate@helmholtz-berlin.de, qiong.wang@helmholtz-berlin.de

**Keywords**: Inorganic perovskite solar cells, surface passivation, air annealing, hard X-ray photoelectron spectroscopy, band bending, transient surface photovoltage

***Experimental section***
***Chemicals***

CsI (99.999%, Sigma Aldrich), lead(II) iodide ($PbI_2$, 99.99%, TCI), dimethyl ammonium iodide (DMAI) (98%, Sigma-Aldrich), methyl ammonium chloride (MACl, Dyenamo), n-octylammonium iodide (OAI, GreatCell Solar), dimethylformamide (DMF, 99.8%, Sigma-Aldrich),iso-propanol (IPA, 99.5%, Sigma-Aldrich),chlorobenzene (99.8%, Sigma-Aldrich), tris(2-(1H-pyrazol-1-yl)-4-tert-butylpyridine)cobalt(III)tri[bis-(trifluoromethane) sulfonimide] (FK209, Dyenamo), bis(trifluoromethane)sulfonimide lithium salt (Li-TFSI, Sigma-Aldrich), ethanol (99.9%, Merck), Spiro-OMeTAD (Lumtec), titanium diisopropoxide bis(acetylacetonate) (TIAP, 75 wt % in isopropanol, Sigma-Aldrich) and 4-tert-butylpyridine (tBP) (98%, Sigma-Aldrich).

All chemicals are used as received.

***F doped tin oxide (FTO) substrates cleaning***

Patterned FTO substrates (TEC 15, Yingkou company, with dimensions 2.5 cm × 2.5 cm) were numbered on the glass side. These numbers were designated to every device like Z1 to Z36. These substrates were cleaned with 2% Mucosal solution using very fine brush to clean FTO surface, then washed with distilled water to remove soap contents. Afterwards, cleaned with acetone for 10 minutes and isopropanol for 15 min by sonication. After drying with a nitrogen gun, the substrates were placed in an UV-ozone cleaner for 20 minutes right before the titanium oxide layer deposition. We have evaporated ~1nm LiF layer on glass.

***Solution preparation***

- 2% Mucasol solution was made by mixing 2 mL mucasol in 100 ml distilled water.
- $TiO_2$ solution was prepared by mixing 150 µL TIAP and 15 mL of ethanol and shaking for 5 minutes.
- 1.5 M $PbI_2$ solution was prepared by dissolving 1.000 g of $PbI_2$ solution in 1.442 ml DMF solvent. The mixture was stirred at 60 °C for 12 hours to get $PbI_2$ solution.
- To make 0.70 M $CsPbI_3$ solution, 1.192 ml $PbI_2$ solution was added in 0.3710 g CsI salt along with 848MicroL solvent and stirred for 10 min at room temperature until it completely dissolved.
- To make 1:1:1 (atomic ratio) CsI: $PbI_2$: DMAI solution, 1.6515 ml above $CsPbI_3$ solution was added in 200 mg DMAI and stirred for 10 min at room temperature until a clear, yellowish perovskite solution was formed.
- 45 mM MACl solution was made by dissolving 30 mg MACl salt in 9.870 mL IPA and was stirred for two hours until it completely dissolved.
- OAI solution was prepared by dissolving 6 mg of OAI in 2mL IPA and stirred for 15 hours.
- A 36.15 mM solution of spiro-OMeTAD was prepared by dissolving 190 mg Spiro-OMeTAD in 2.1 ml chlorobenzene with 83.39 µL tBP, 48.46 µl LiTFSI with stock solution of 520 mg/ml in acetonitrile, and 21.11 µl FK209 with stock solution of 375 mg/ml in acetonitrile.

***Device Fabrication***

***$TiO_2$ compact layer***

$TiO_2$ compact layer ($TiO_2$-C) was deposited by spray pyrolysis method in which oxygen was used as the carrier gas. In a batch 16 substrates were placed on a hot plate installed inside a fume hood. One rectangular side of each substrate is covered by around 4 mm using a cover glass to keep the conductive FTO side exposed for low contact resistance. Then the substrates were heated up to 450 °C and were kept at this temperature for 10 min before and 30 min after the precursor's spray. The whole solution was transferred into a spray nozzle and sprayed at roughly 20-25 cm away from the substrates with an inclination angle of 45 degree, with at least 15 s of delay between each spraying cycle. Afterwards, substrates were left for cooling down to 150°C temperature and then put in an ozone chamber for 15 minutes before perovskite film deposition.

***Deposition of control perovskite films***

After 20 minutes ozone treatment, substrates were transferred into a glove box filled with nitrogen ($O_2$ <0.1 ppm, $H_2O$ <0.1 ppm). The substrates were placed on a hot plate at 60 °C for 5 min before perovskite deposition. 80-100 µl perovskite solution was added and spin-coated quickly at 3000 rpm for 30 seconds. Then 350 µl MACl solution was dropped on the top and spin coated for another 35 seconds. The wet films were then annealed in a dry air box with a relative humidity (RH) of ~ 1% for 1 min at 210 °C and other films were annealed in air in fume hood at 210°C. Afterwards, substrates were transferred back to a nitrogen-filled glove box, where 100 µl OAI solution was dropped on the top and spin coated at 5000 rpm for 30 seconds, followed by annealing at 100 °C for 5 min.

***Hole Transport Material (HTM) Layer deposition***

100 µl Spiro-OMeTAD solution was deposited by spin coating at 3500 rpm for 35 seconds. No annealing is needed for this step.

Afterwards, all the samples were transferred into a dry air box (RH ~ 0.1%) for oxygen soaking for overnight.

***Deposition of metal contact***

Gold was evaporated using a thermal evaporator under the vacuum of approximately $2*10^{-6}$ pa. The deposition rate was programmed at 0.015 Å/s for the first 1 nm, 0.1-0.2 Å/s for the following 5 nm, and then 0.5 Å/s until 20 nm and then 1Å/s for the rest of the deposition. Overall, it takes around 30 min for the deposition of 100 nm of gold. The active area of the device was 0.18 $cm^2$ defined by the shallow mask.

***Solar cell characterization***

The light source was provided by an Oriel LCS-100 class ABB solar simulator (1Sun, AM1.5G, 100 $mWcm^{-2}$) installed inside a nitrogen-filled glovebox. Before the light *J-V* measurement, the light intensity was calibrated with a silicon reference cell (Fraunhofer ISE). A Keithley power meter (2400 SMU) was used for the bias application to solar cells for the *J-V* scans, programmed by the LabView. The bias was applied to scan from 1.30 V to -0.1 V reverse scan with a scan rate of 200 mV/s and a step size of 0.02 V.

***External Quantum efficiency (EQE) measurements***

EQE spectra were recorded with the TracQ-Basic software, connected to the light source (Oriel Instruments QEPVSI-b system integrated with a Newport 300 W xenon arc lamp) with an optical fiber. The spectrum of the light source was calibrated with a Si reference cell with known spectral response before the measurement. The monochromatic light was provided by a Newport Cornerstone 260 monochromator with a chopping frequency of 78 Hz.

***Scanning electron microscope (SEM)***

The SEM images were recorded with the Hitachi S-4100 at an acceleration voltage of 5 kV.

***UV-vis spectroscopy***

Perkin Elmer LAMBDA 1050 UV/VIS spectrometer was used in transmittance mode.

***Stead State Photoluminescence Spectroscopy (PL)***

A 445 nm CW laser (Insaneware) was used as the excitation source for the PL measurements with an optical fiber connected to an integrating sphere where samples were loaded. Samples for the PL measurements were encapsulated with a cover glass before being taken out of a nitrogen-filled glovebox.

***Time-resolved photoluminescence spectroscopy (TRPL)***

Time-resolved PL data was acquired with a TCSPC system (Berger & Lahr) after excitation with a mode-locked Ti:sapphire oscillator (Coherent Chameleon) that provides a pulse-picked and frequency-doubled output, with nominal pulse durations ~ 100 fs and fluence of ~30 nJ/cm$^2$ at a wavelength of 470 nm.

***Hard X-ray Photoelectron Spectroscopy measurements***

HAXPES measurements were conducted at the HiKE endstation located at the BESSY II KMC-1 beamline at Helmholtz-Zentrum Berlin für Materialien und Energie GmbH (HZB). [1,2] The endstation is equipped with a Scienta R4000 electron analyzer, allowing it to use of the excitation energy range (i.e., 2 – 10 keV) provided by the KMC-1 bending magnet beamline. Two different excitation energies are employed in this work, making use of different diffraction orders of the Si (111) crystal pair of the KMC-1 double crystal monochromator (i.e., 2 keV in 1st order and 6 keV in 3rd order); with these excitations, it is possible to probe the topmost and buried layers of approximately 12 and 30 nm, respectively.[3,4] The energy scale of the HAXPES measurements was calibrated using Au 4f reference spectra of a clean Au foil, setting the BE of the Au $4f_{7/2}$ line to 84.00 eV. Measurement protocols involving the use of a beam-attenuating Be filter (that effectively reduces the 2 keV photon flux by 75% and the 6 keV photon flux by 10% of their unfiltered values) was implemented in this experiment, aiming at preventing/minimizing beam-induced damage to the sample. Moreover, we applied a segmented data acquisition approach to compare the evolution of core levels under X-ray radiation and move sample spots. Curve fit analysis of the measured detail HAXPES spectra were simultaneously conducted with the Fityk software [5]. Voigt profile functions and linear backgrounds were used for these fits. Spin-orbit doublets were fit using two Voigt functions

with intensity ratios set to obey the 2$j$+1 multiplicity rule. HAXPES-derived [Cs]:[Pb]:[I] composition ratio quantifications were carried out by correcting the peak intensities of the Cs $4d_{5/2}$, Pb $5d_{5/2}$ and I $4d_{5/2}$ HAXPES shallow core levels to account for differences in photoionization cross section.[(6-8)] Due to the energetic proximity of these lines, the impact of differences in inelastic mean free path (IMFP) [(3,4)] and the transmission function of the electron analyzer [(9)] on the intensity of the core levels is negligible.

### *Kelvin probe measurement for work function*

The measurement of WF was performed by non-contact and non-destructive Kelvin probe method, in which the sample and probe form a parallel plate capacitor.

### *Transient surface photovoltage (tr-SPV) measurements.*

Charge extraction in the time range of 5 ns up to 0.5 s was studied by non-contact SPV measurements exited by 5 ns above bandgap laser (1.8 eV). We used fluences of 0.072 µJ, which corresponds to a carrier concentration of $3\times10^{15}$ $cm^{-3}$ close to 1 sun operation conditions. Detailed SPV setup description is given in our previous study. [(10,11)] Contour plots were recorded with a tunable laser in the range 0.6-3 eV using fluences of 72 µJ to ensure a good signal to noise ratio.

### *Simulation of charge extraction and recombination*

**Eq. S1-5** describe the simulation model for charge separation, trapping, and recombination where $n$ and $p$ are the concentration of photo-induced electrons and holes. The constants $K_e$ and $K_h$ correspond to electron and hole injection rates from perovskite to HTM. [(11,12)] The constant $K_{eTiO}$ corresponds to electron injection rates from perovskite to ETM ($TiO_2$). Similarly, $K_{eb}$ and $K_{hb}$ are reinjection rates of electron and hole to perovskite from HTM, which effectively include back tunnelling/thermionic emission, and diffusion of the free carriers, as well as the drift of the free carriers due to the presence of the space charge. $C_b$is radiative recombination constant. $N_t$ and σ are concentration and capture cross-section of defect responsible for SRH non-radiative recombination. $N_{St}$ and $\sigma_{Se/h}$ are concentrations and capture cross-section of defects responsible for SRH non-radiative recombination at the perovskite surface. The system of the equations cannot be solved analytically; therefore, we used the Adams backward differentiation formula (BDF) solving algorithm. We used the Levenberg-Marquardt method to fit constants with minimal deviation from experimental SPV results. SPV data were extrapolated logarithmically for better fitting results. The results of the fit are given in **Fig.4a** and summarized in **Table S4**.

$$\frac{dp}{dt} = -K_h p + K_{hb} p_{HTM} - C_b(pn) - p\sigma_{ht} v_h n_t - p\sigma_{Sht} v_h n_{St} \qquad \text{(Eq. S1)}$$

$$\frac{dn}{dt} = -K_e n + K_{eb} n_{HTM} - C_b(np) - n\sigma_{et} v_e (N_t - n_t) - K_{eTio} n - n\sigma_{Set} v_e (N_{St} - n_{St}) \qquad \text{(Eq. S2)}$$

$$\frac{dp_{HTM}}{dt} = K_h p - K_{hb} p_{HTM} \qquad \text{(Eq. S3)}$$

$$\frac{dn_{HTM}}{dt} = K_e n - K_{eb} n_{HTM} \qquad \text{(Eq. S4)}$$

$$\frac{dn_{ETM}}{dt} = K_{eTio} n \qquad \text{(Eq. S5)}$$

$$\frac{dn_t}{dt} = n\sigma_{et}v_e(N_t - n_t) - p\sigma_{ht}v_h n_t \qquad \text{(Eq. S6)}$$

Due to $d_{PER} \gg d_{HTM}$, $d_{PER} \gg d_{ETM}$, and assuming a uniform distribution of charges in perovskite, ETM, and HTM layers; so SPV signal can be simplified in the form:

$$SPV = \frac{L}{2}\frac{n_{ETM}}{\varepsilon_{TiO}\varepsilon_0} + \frac{L}{2}\frac{n_{HTM} - dp_{HTM}}{\varepsilon_{HTM}\varepsilon_0} + \frac{L}{2}\frac{n-p-nt}{\varepsilon_{pero}\varepsilon_0} \qquad \text{(Eq. S7)}$$

Where $L = d_{PER}/2$ -charge separation distance.

***Ageing of Solar Cells (long-term stability measurement)***

Solar cells were aged in a custom-built High-throughput Ageing Setup. [13] A light-cycling experiment according to ISOS-LC-1I [14] was performed with constant illumination. During the illumination phase, special electronics were used to MPP-track all cells individually. A perturb and observe algorithm [15] with a delay time of 1 s and a voltage step-width of 0.01 V was applied to track the MPP. $PCE_{MPP}$ values were taken every 2 min for all cells automatically.

Additionally, *JV*-scans, with a scan speed of 90 mV/s, were performed on every cell after 11 hours of the light phase of a cycle. During the dark phase, the shutter was shortly opened to perform *JV*-scans on selected pixels after 11.5 hours of darkness.

Devices were always kept at 25 °C with the help of actively controlled Peltier elements. Solar cells' active areas were touching a heating pad for direct thermal coupling. Aging was performed under a continuous flow of nitrogen in a closed box, no additional encapsulation was used. Sunlight with 1 sun intensity was provided by a metal-halide lamp using a H6 filter. A UV-blocking foil was used to block UV light with wavelengths below 380 nm. **Fig. S19** shows the spectrum of the light source in comparison to AM1.5G. The light intensity was actively controlled with the help of a silicon irradiation-sensor which was calibrated using a KG3 silicon reference cell from Fraunhofer ISE.

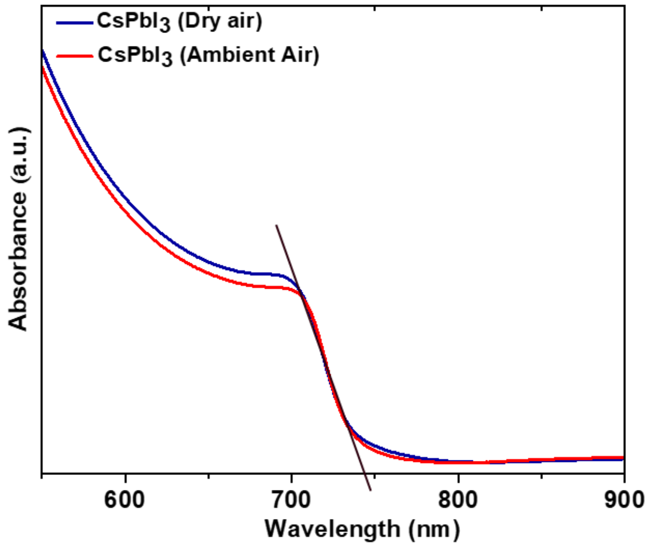


**Figure S1** Absorbance spectra of dry air and ambient air annealed $CsPbI_3$ absorbers measured by UV-Vis spectroscopy.

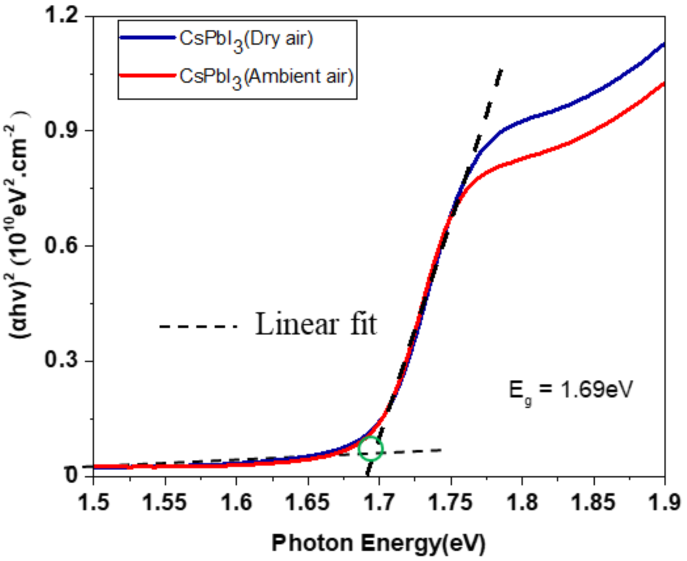


**Figure S2**. Tauc plot of the absorbance (presented in (**Figure S1**) derived for the differently annealed $CsPbI_3$ absorbers

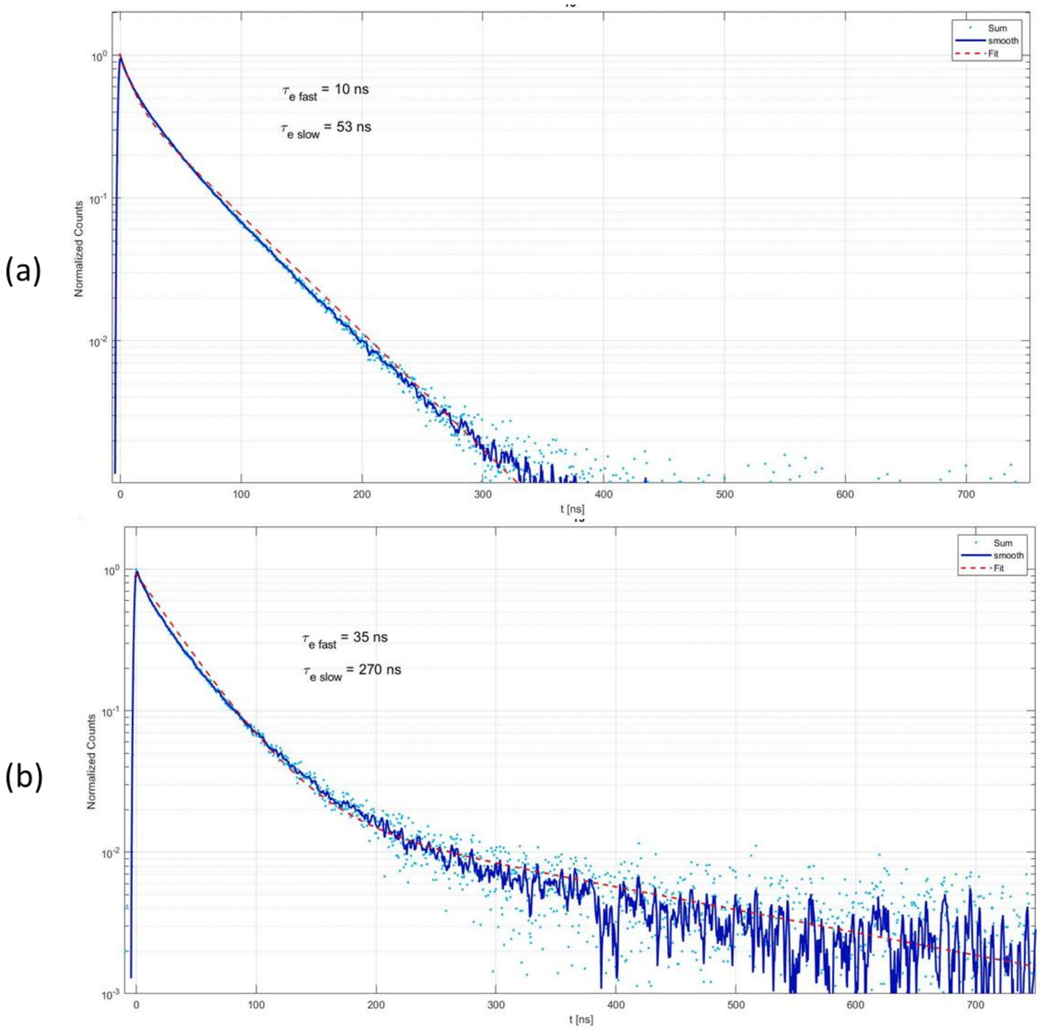


**Figure S3** TRPL spectroscopy measured for a) dry air and b) ambient air annealed samples

Transient Photoluminescence (TRPL) spectra were fitted with the bi-exponential model as given by the following relation:

$$I_{PL}\,(t) = A\exp\left(-\frac{t}{\tau_{fast}}\right) + B\exp\left(-\frac{t}{\tau_{slow}}\right) \qquad \textbf{Eq S8}$$

We attribute the initial exponential decay ($t_1$) to charge transfer to $TiO_2$ and the second exponential decay $t_2$, to non-radiative interfacial recombination recombination. [11,16] We note, due to the influence of charge extraction on the transients no average lifetime is caluated.

**Table S1:** Fitted parameters of trPL data

| Sample | $\tau_1$(ns) | $\tau_2$ (ns) |
|---|---|---|
| Glass/$TiO_2$/$CsPbI_3$: OAI (dry air annealed) | 10 | 53 |
| Glass/$TiO_2$/$CsPbI_3$: OAI (ambient air annealed) | 35 | 270 |

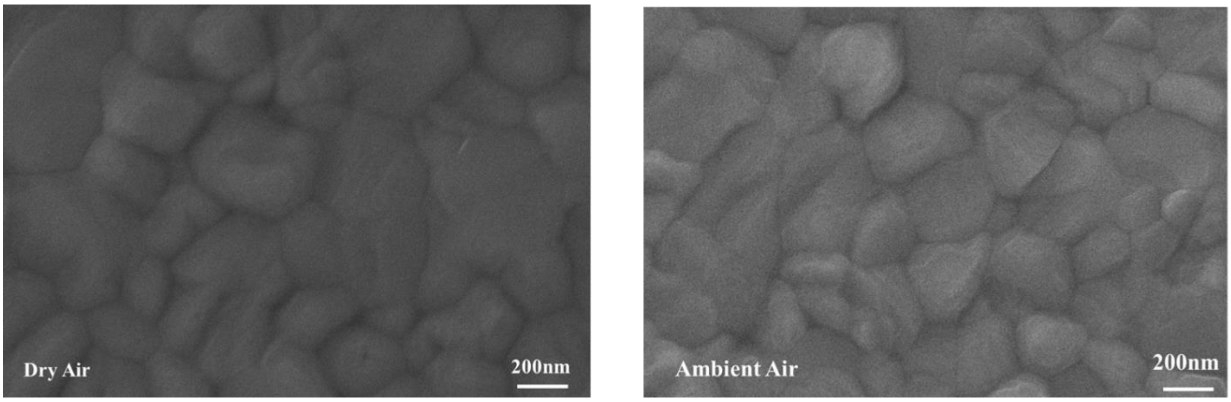


**Figure S4.** Top view SEM image of the (a) dry air annealed (b) ambient air annealed film spin coated on compact $TiO_2$ layer and FTO substrates**.**

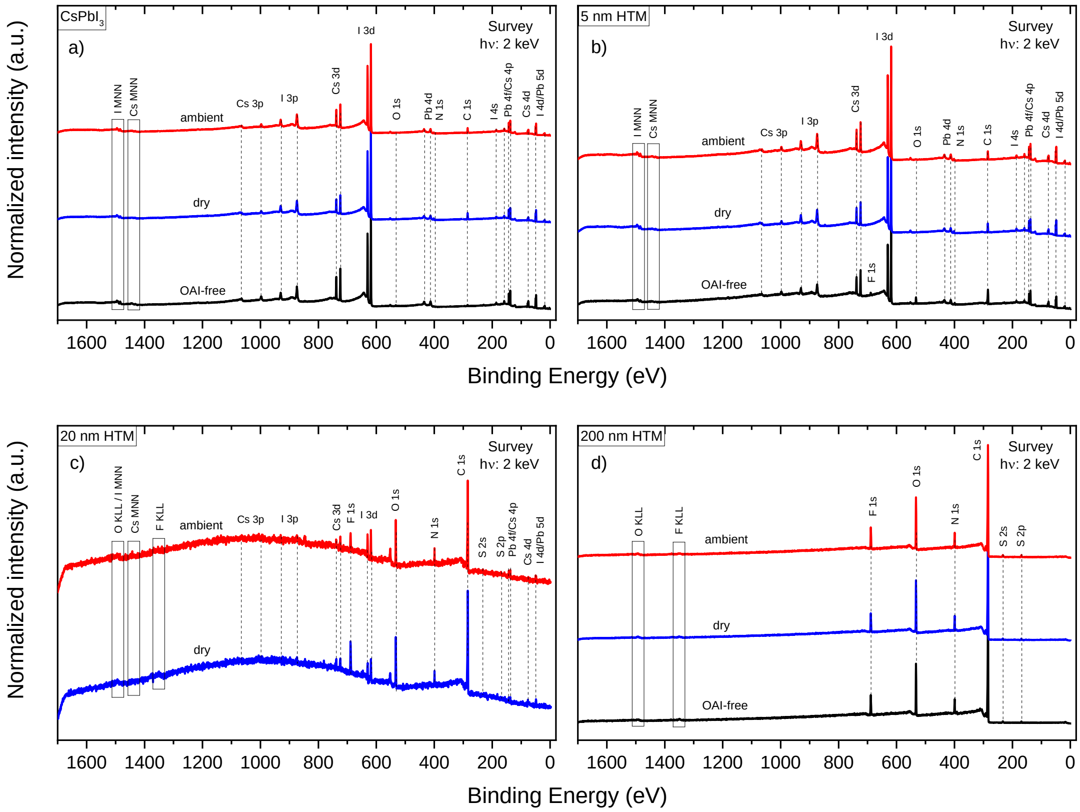


**Figure S5** Hard X-ray photoelectron spectroscopy (HAXPES) survey spectra of variously treated $CsPbI_3$ films (i.e., OAI-free (dry air annealed), dry air annealed and ambient air annealed) with a) 0 nm (i.e., bare), b) 5 nm, c) 20 nm and d) 200 nm films of spiro-OMeTAD, used as a hole transport material (HTM). In samples with 20 nm and 200 nm layers of HTM, there is a clear signal from F and S, indicating significant contribution from LiTFSI – the F signal may also be related to FK 209. The spectra were measured using 2 keV excitation and normalized to background intensity, with vertical offsets added for clarity.

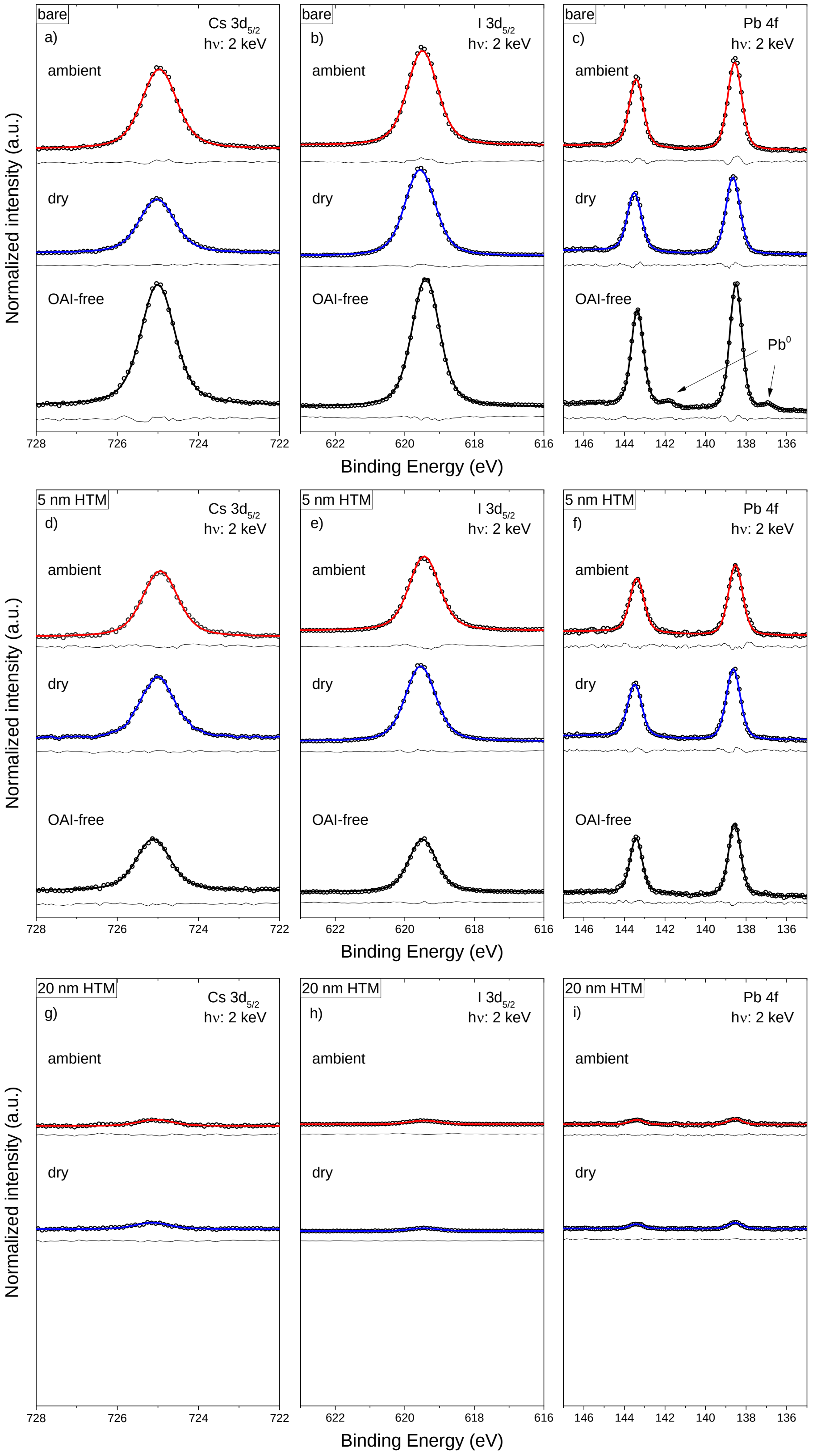


**Figure S6** HAXPES detail spectra of the Cs $3d_{5/2}$ (a, d, g), I $3d_{5/2}$ (b, e, h) and Pb 4f (c, f, i) photoemission lines for the variously treated $CsPbI_3$ films (i.e., OAI-free, dry air annealed and ambient air annealed) with 0 nm (i.e., bare), 5 nm and 20 nm films of Spiro-OMeTAD, respectively. The spectra were measured using 2 keV excitation and normalized to background intensity, with vertical offsets added for clarity. Curve fit results are included.

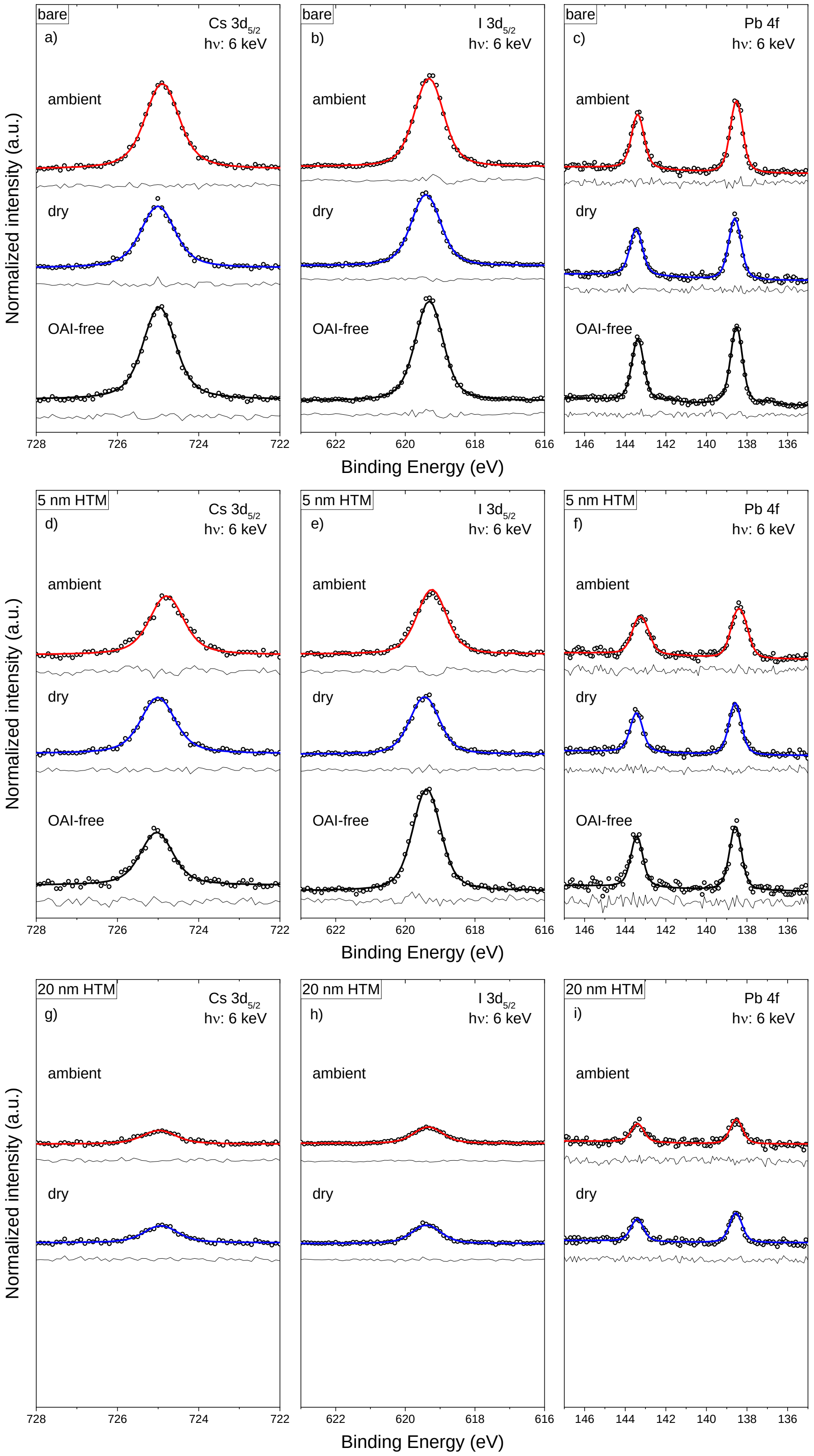


**Figure S7** HAXPES detail spectra of the Cs $3d_{5/2}$ (a, d, g), I $3d_{5/2}$ (b, e, h) and Pb 4f (c, f, i) photoemission lines for the variously treated $CsPbI_3$ films (i.e., OAI-free, dry air annealed and ambient air annealed) with 0 nm (i.e., bare), 5 nm and 20 nm films of spiro-OMeTAD, respectively. The spectra were measured using 6 keV excitation and normalized to background intensity, with vertical offsets added for clarity. Curve fit results are included.

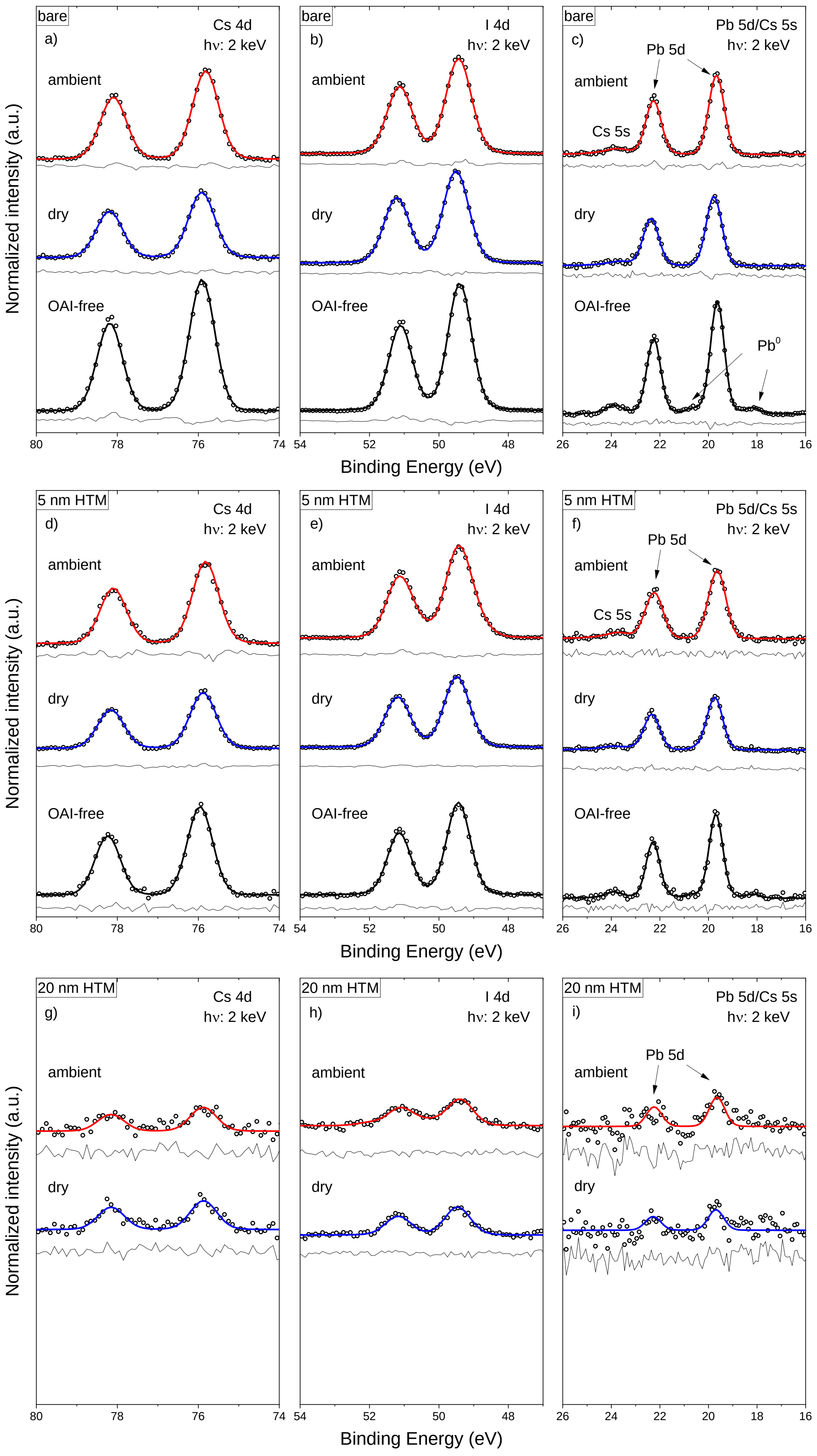

bare
a)
Cs 4d
hν: 2 keV
ambient
dry
OAI-free
b)
I 4d
c)
Pb 5d/Cs 5s
Pb 5d
Cs 5s
Pb0
5 nm HTM
d)
e)
f)
20 nm HTM
g)
h)
i)
Normalized intensity (a.u.)
Binding Energy (eV)
80
78
76
74
54
52
50
48
26
24
22
20
18
16

**Figure S8** HAXPES detail spectra of the Cs 4d (a, d, g), I 4d (b, e, h) and overlapping Pb 5d/Cs 5s (c, f, i) photoemission lines for the variously treated $CsPbI_3$ films (i.e., OAI-free, dry air annealed and ambient air annealed) with 0 nm (i.e., bare), 5 nm and 20 nm films of spiro-OMeTAD, respectively. The spectra were measured using 2 keV excitation and normalized to background intensity, with vertical offsets added for clarity. Curve fit results are included.

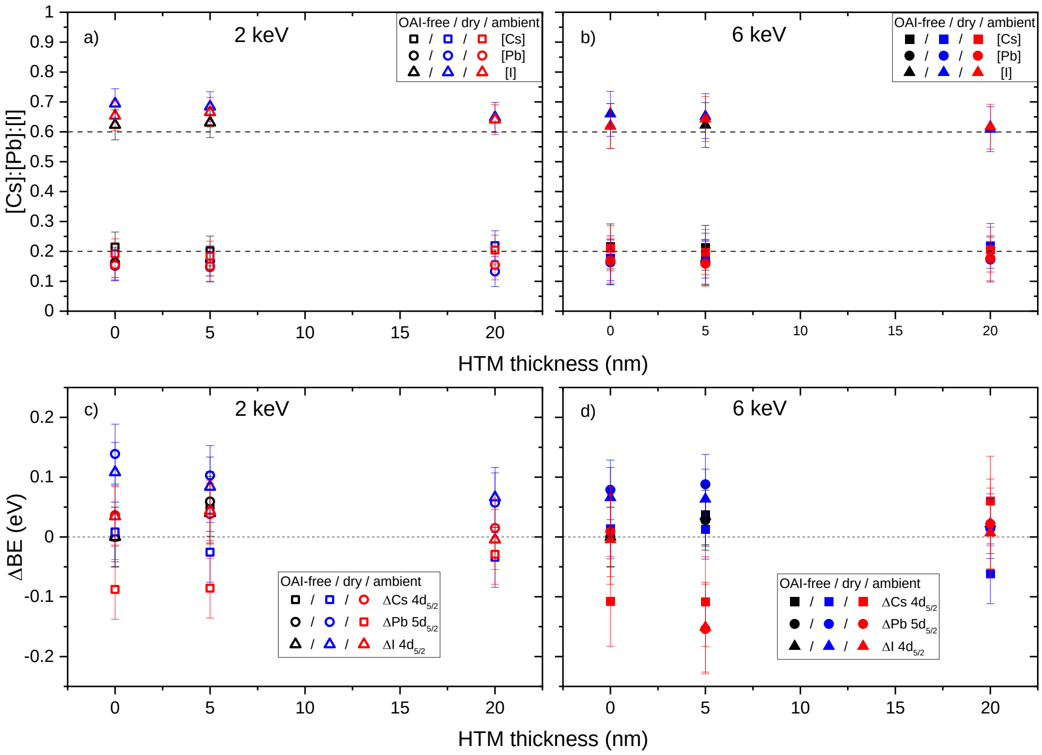


**Figure S9.** [Cs]: [Pb]: [I] surface composition of variously treated $CsPbI_3$ films (i.e., OAI-free, dry air and ambient air annealed) with 0 nm (i.e., bare), 5 nm and 20 nm films of HTM, determined by HAXPES measurements employing a) 2 keV and b) 6 keV excitations. Horizontal dashed lines are added to show a [Cs]: [Pb]: [I] composition ratio of 1:1:3 ratio, the nominal stoichiometry of the $CsPbI_3$ samples. Changes in binding energy (BE) values of the HAXPES Cs $4d_{5/2}$, I $4d_{5/2}$, and Pb $5d_{5/2}$ peaks, measured with excitation energies of c) 2 keV and d) 6 keV (derived from the spectra shown in **Figs. S6 and 2**), compared to the corresponding peak BE values of the bare, OAI-free $CsPbI_3$ sample.

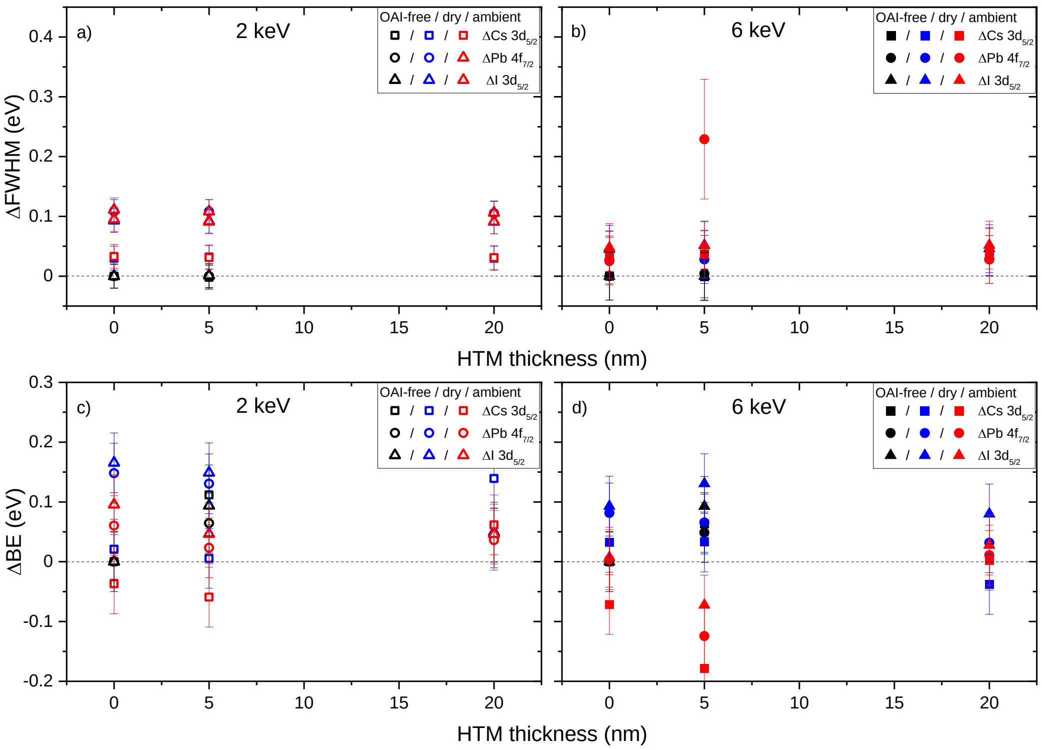


**Figure S10** Changes in full-width-half-maximum (FHWM) values of the HAXPES Cs $3d_{5/2}$, I $3d_{5/2}$ and Pb $4f_{7/2}$ peaks, measured with a) 2 keV and b) 6 keV excitations, of the investigated samples (shown in **Figs. S7** and **S8**), compared to peak FHWM values of the bare, OAI-free $CsPbI_3$ sample. Changes in BE values of the HAXPES Cs $3d_{5/2}$, I $3d_{5/2}$, and Pb $4f_{7/2}$ peaks, measured with excitation energies of c) 2 keV and d) 6 keV, compared to the corresponding peak BE values of the bare, OAI-free $CsPbI_3$ sample.

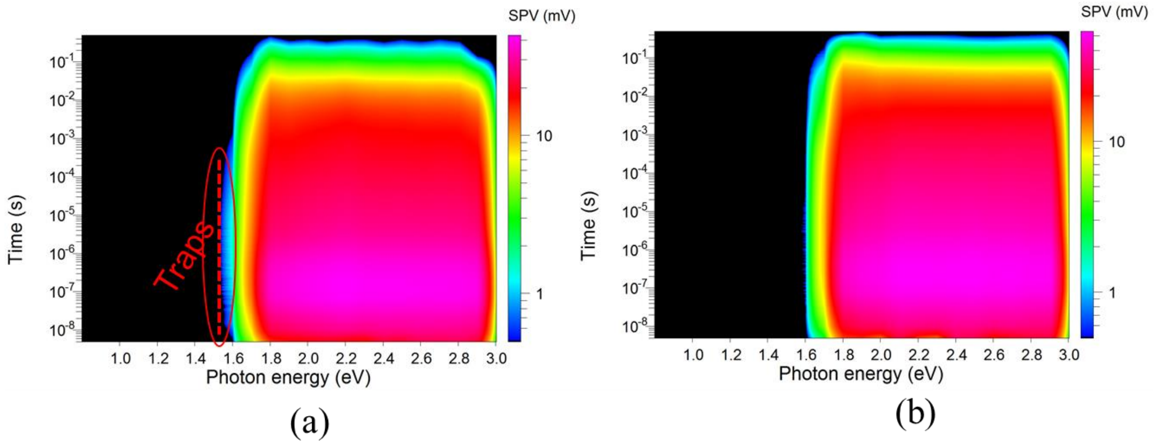


**Figure S11** Contour plots of spectral dependence transient SPV mapping of a) glass/$TiO_2$/$CsPbI_3$/OAI (dry air annealed) b) glass/$TiO_2$/$CsPbI_3$/OAI (ambient air).

**Figure S11** shows tr-SPV signals as a function of photon energy (eV) and time (seconds). Whereas the bar code on the right corner presents the tr-SPV signals amplitude. The SPV signals are recorded with laser light excitation by photon energy in the range of 0.7-3 eV. The excitation of the traps by excitation light leads to charge separation. The signal below the band gap corresponds to the activation energy of the trap. [17,18,19] Here, we observe very few traps below the band gap encircled in Figure **S11 a**. However, we do not see these traps in ambient air annealed samples as shown in Figure **S11b**.

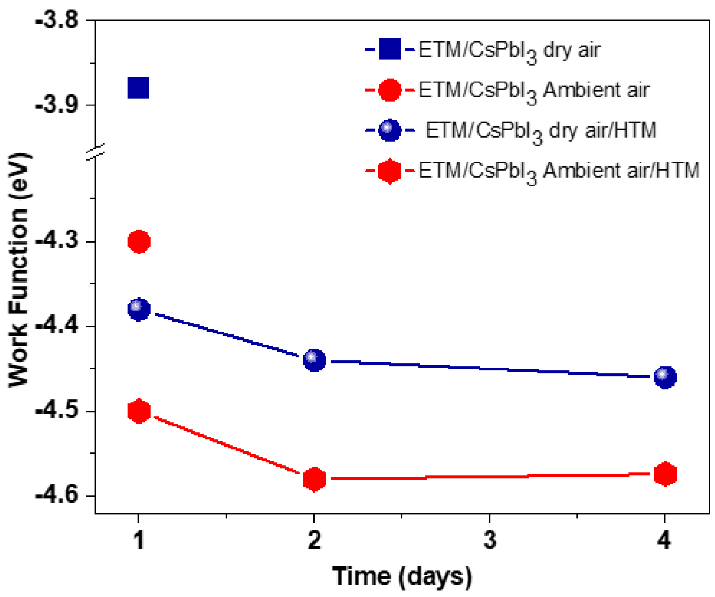


**Figure S12** Work function measurements with Kelvin Probe method for dry air annealed and ambient air annealed samples.

**Figure S12** shows a work function change for spiro-OMeTAD capped dry and ambient air annealed samples for up to four days. The time tracking shows that more accelerated increase in WF in the ambient air-annealed samples. The effect of this WF change has been discussed in the device performance section.

**Table S2** Work function values (in eV) for dry air annealed and ambient air annealed samples for **Figure S12**

| **Sample** | **Day1** | **Day2** | **Day4** |
|---|---|---|---|
| $TiO_2/CsPbI_3$ dry air | 3.88 | | |
| $TiO_2/CsPbI_3$ ambient air | 4.30 | | |
| $TiO_2/CsPbI_3$ dry air/HTM | 4.50 | 4.58 | 4.57 |
| $TiO_2/CsPbI_3$ ambient air/HTM | 4.38 | 4.44 | 4.46 |

**Table S3. Additional constants for simulations**

Where *N, P,* $C_b$, $\sigma_e$, $\sigma_h$, $v_e$, and $v_h$ are photo-induced electron concentration, photo-induced hole concentration, radiative recombination constant, electron capture-cross section of trap, hole capture cross-section of trap, thermal velocity of electron and hole, respectively. $\varepsilon_{per}$, $\varepsilon_{ETM}$ and $\varepsilon_{HTM}$ are dielectric constants. The concentration of free carriers (*N, P*) was calculated according to the fluence of 0.10 μJ/cm$^2$. Constants *C*b, $v_e$, $v_h$, $\boldsymbol{\sigma_{Se/h}}$ and $\boldsymbol{\varepsilon_{per}}$, were adapted from our previous study (10,11). Dielectric constants $\varepsilon_{ETM}$ and $\varepsilon_{HTM}$ were adapted from literature. (20,21) Constants $K_{eETL}$, $\sigma_e$, and $\sigma_h$ are fitted directly and are in agreement with previous reports (19,22,23)

| ***N* (P), cm$^{-3}$** | **$C_b$, cm$^3$s$^{-1}$** | **$K_{eETL}$, 10$^7$ s$^{-1}$** | **$\sigma_e$, cm$^2$** | **$\sigma_h$, cm$^2$** | **$v_e$, cms$^{-1}$** |
|---|---|---|---|---|---|
| $1.1\times10^{15}$ | $5\times10^{-10}$ | 1.2-1.8 | $10^{-13}$ | $4\times10^{-14}$ | $3\times10^{7}$ |
| ***$v_h$, cm s$^{-1}$*** | *$\varepsilon_{per}$* | *$\varepsilon_{ETM}$* | *$\varepsilon_{HTM}$* | | |
| $3\times10^{7}$ | 18 | 3 | 3 | | |

**Table S4** Main fitting constants of simulation. $N_{St}$ – concentration of surface defects inducing non-radiative recombination of carriers, $K_h$ – hole injection rate, STD is the average standard deviation of the fit from the experimental SPV signal.

| Interface ($TiO_2$/$CsPbI_3$/HTM) | $K_h$, S$^{-1}$ | $N_{st}$, cm$^{-3}$ | STD, % |
|---|---|---|---|
| Dry air annealed | $2.4\times10^6$ | $5.6\times10^{14}$ | 16 |
| Ambient Air annealed | $6.1\times10^6$ | $3.7\times10^{11}$ | 7 |

**Table S5** Champion devices performance parameters for dry air and ambient air annealed samples

| Device | $J_{SC}$ (mA/cm$^2$) | $V_{OC}$ (Volts) | *FF* (%) | PCE (%) |
|---|---|---|---|---|
| Dry air (for) | 20.2 | 1.11 | 73.6 | 16.5 |
| Dry air (rev) | 20.2 | 1.18 | 77.7 | 18.6 |
| Ambient air (for) | 20.4 | 1.15 | 73.8 | 17.2 |
| Ambient air (rev) | 20.4 | 1.23 | 78.9 | 19.8 |

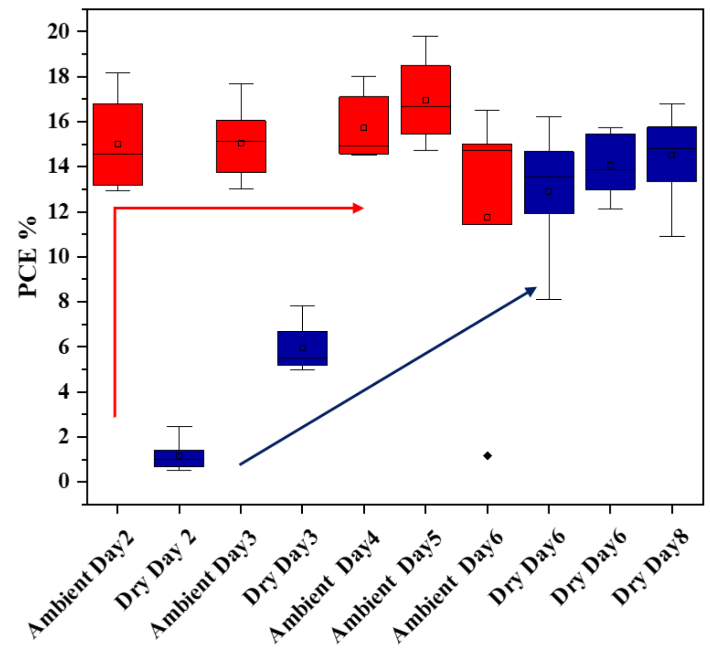


**Figure S13** PCE measured for dry air and ambient air annealed samples-based devices soaked on different days.

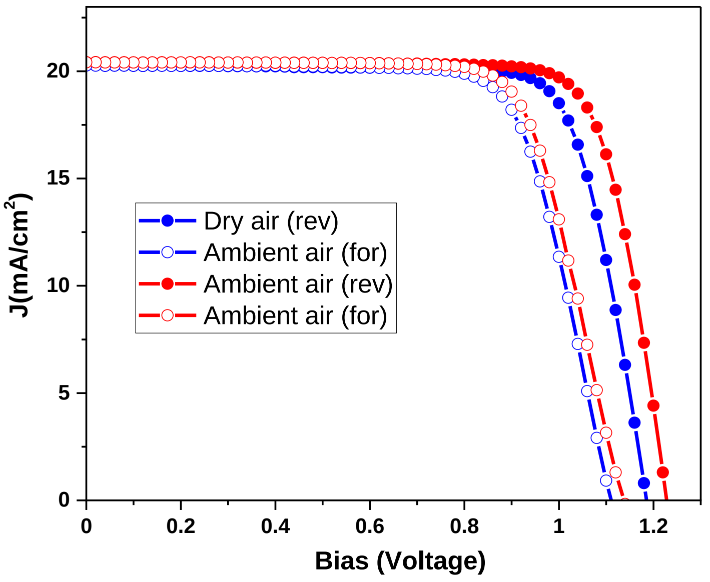


**Figure S14**. Champion devices performance parameters for dry air and ambient air annealed samples (forward and reverse scan)

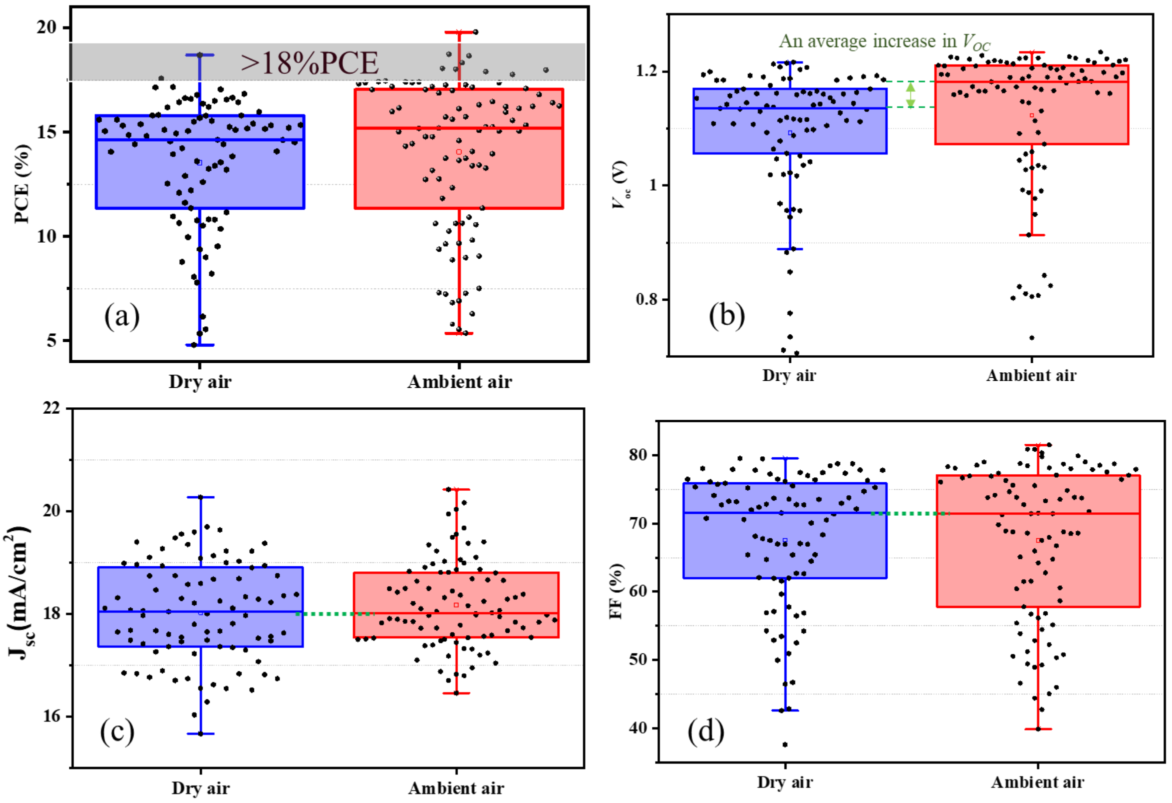


**Figure S15:** Box chart of 18 dry air and 18 ambient air annealed films based individual devices with a total over 90 pixels showing corresponding (a) PCE %, (b) Open circuit voltage, $V_{OC}$, (c) Current density, $J_{SC}$, and (d) Fill factor, *FF*, values.

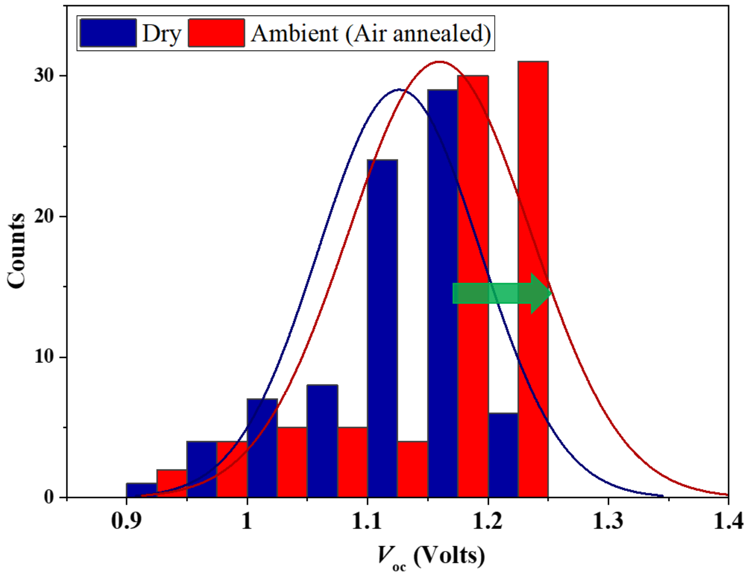


**Figure S16** Histogram of $V_{OC}$ for dry and ambient air-annealed $CsPbI_3$ film-based devices.

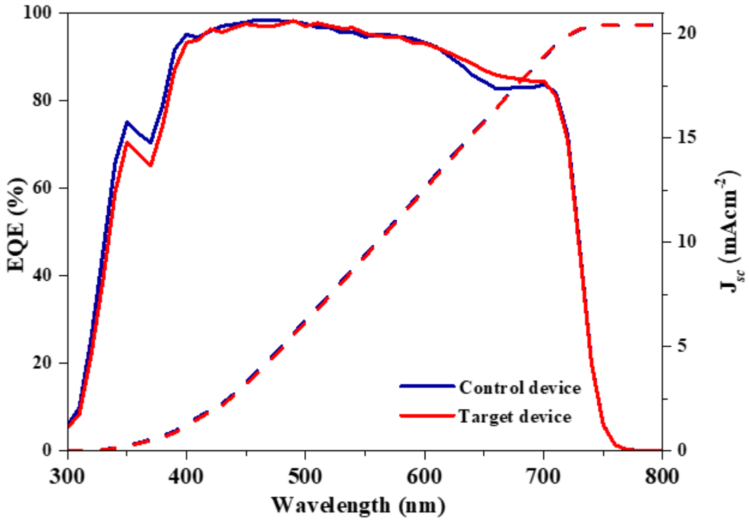


**Figure S17** External quantum efficiency (EQE) measurements for the champion dry- and ambient-air annealed $CsPbI_3$ absorber devices.

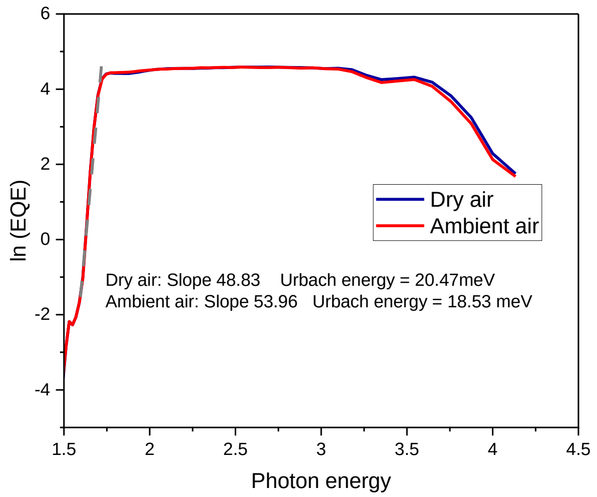


**Figure S18.** Urbach energy ($E_0$) of dry air and ambient air annealed films. $E_0$ is calculated from EQE measurements.

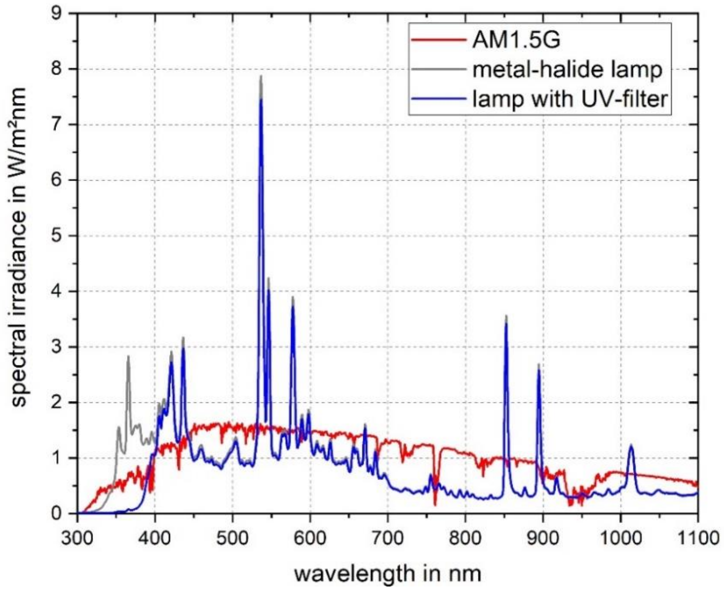


**Fig. S19**. Spectrum of the lamp of the High-throughput Ageing Setup used to age solar cells in comparison to AM1.5G. In this work, a UV-filter was used (blue curve).

## References


1. M. Gorgoi, S. Svensson, F. Schäfers, G. Öhrwall, M. Mertin, P. Bressler, O. Karis, H. Siegbahn, A. Sandell, H. Rensmo, W. Doherty, C. Jung, W. Braun, W. Eberhardt., "The high kinetic energy photoelectron spectroscopy facility at BESSY progress and first results." *Nuclear Instruments and Methods in Physics Research Section A: Accelerators, Spectrometers, Detectors and Associated Equipment*, 2009, **601**, 48-53.
2. F. Schaefers, M. Mertin, and M. Gorgoi., "KMC-1: A high resolution and high flux soft x-ray beamline at BESSY.", *Rev Sci Instrum*, 2007,**78**, 123102.

3. S. Tanuma, C. J. Powell and D. R. Penn., “Calculations of electron inelastic mean free paths. V. Data for 14 organic compounds over the 50–2000 eV range.” *Surface and interface analysis,* 1994, **21**,165-176
4. S.Tougaard, QUASES-IMFP-TPP2M Program, Quases-Tougaard Inc., Odense, Denmark, 2002.
5. M.Wojdyr., “Fityk: a general-purpose peak fitting program.”, *J. Appl. Crystallogr.*2010, **43**, 1126.
6. M.B,Trzhaskovskaya,V.I.Nefedov, V.G. Yarzhemsky., “Photoelectron Angular Distribution Parameters for Elements Z=1 to Z054 in the photoelectron Energy range100-5000 eV.”, At. Data Nucl. Data Tables,2001, 77, 97–159.
7. M.B,Trzhaskovskaya,V.I.Nefedov, V.G. Yarzhemsky., “Photoelectron Angular Distribution Parameters for Elements Z=1 to Z=54 in the photoelectron Energy range100-5000 eV.”, *At. Data Nucl. Data Tables*,2002,**82**, 257–311.
8. M.B,Trzhaskovskaya,V.I.Nefedov, V.G. Yarzhemsky., “Photoelectron Angular Distribution Parameters for Elements Z=1 to Z=54 in the photoelectron Energy range100-5000 eV.”, *At. Data Nucl. Data Tables*,2006,**92**, 245-304.
9. M.P. Seah., “A system for the intensity calibration of electron spectrometers.”, *Journal of Electron Spectroscopy and Related Phenomena*,1995,**71**,191-204.
10. Z. Iqbal, F. Zu, A. Musiienko, E. Gutierrez-Partida, H. Köbler, T. W. Gries, *et al.*, “Interface Modification for Energy Level Alignment and Charge Extraction in $CsPbI_3$ Perovskite Solar Cells.” *ACS Energy Letters*,2023, **8**, 4304-4314
11. I. Levine, A. Al Ashouri, A. Musiienko, *et al.* “Charge transfer rates and electron trapping at buried interfaces of perovskite solar cell”, *Joule*, 2021, **5**, 2915–2933.
12. K. Prashanthan, I. Levine, A. Musiienko, E. Gutierrez-Partida, H. Hempel, K. Lips, *et al.*, “Internal electric fields control triplet formation in halide perovskite-sensitized photon upconverters.”, *iScience*, 2023, **26**(4).
13. H. Köbler, S. Neubert, M. Jankovec, *et al.* “High- Throughput Aging System for Parallel Maximum Power Point Tracking of Perovskite Solar Cells”, *Energy Technol.* 2022, **10**, 2200234
14. M.V. Khenkin, E.A. Katz, A. Abate, *et al.*, “Consensus statement for stability assessment and reporting for perovskite photovoltaics based on ISOS procedures”, *Nat. Energy*, 2020, **5**, 35–49.
15. L. Rakocevic, F. Ernst, N. T. Yimga, *et al.* “Reliable Performance Comparison of Perovskite Solar Cells Using Optimized Maximum Power Point Tracking”, *Solar RRL,* 2019, **3**, 1800287.
16. B.Yu, J. Shi, S. Tan, Y.Cui, W. Zhao, H. Wu, Y.Luo, D. Li, Q.Meng. ,“Efficient (>20%) and Stable All-Inorganic Cesium Lead Triiodide Solar Cell Enabled by Thiocyanate Molten Salts” *Angew. Chem. Int. Ed.* 2021, **60**, 13436 – 13443.
17. S. Fengler, T. Emmler, C. Wolpert, M. Schieda, M. Villa Vidaller, T. Klassen, et al., “Influence of Surface States and Mobility on Charge Transport in BiVO4 Investigated by Surface Photovoltage Spectroscopy.” ECS Meeting Abstracts,2020, MA2020-01 I **(39)**,1757, DOI: 10.1149/MA2020-01391756mtgabs
18. S. Fengler, H. Kriegel, M. Schieda, H. Gutzmann, T. Klassen, and T. Dittrich, “Defects Near c-Si(n++)/TiO2 Interfaces Revealed by Persistent Charging Analysis in

Modulated Surface Photovoltage Spectroscopy.” ECS Meeting Abstracts,2020, MA2020-01(**39**) ,1756, DOI: 10.1149/MA2020-01391756mtgabs
19. A. Musiienko, D. R. Ceratti, J. Pipek, *et al*., “Defects in Hybrid Perovskites: The Secret of Efficient Charge Transport”, *Adv. Funct. Mater*., 2021, **31**, 2104467
20. Y. C. Lin, L. Y. Chen, & F. C. Chiu, “Lossy Mode Resonance-Based Glucose Sensor with High-κ Dielectric Film”, *Cryst*., 2019, **9**, 450.
21. F. Anwar, R. Mahbub, S. S. Satter, *et al*., “Effect of Different HTM Layers and Electrical Parameters on ZnO Nanorod-Based Lead-Free Perovskite Solar Cell for High-Efficiency Performance”, *Int. J. Photoenergy*, 2017, DOI: 10.1155/2017/9846310.
22. A. Musiienko, J. Pipek, P. Praus, *et al*., “Deciphering the effect of traps on the electronic charge transport properties of methylammonium lead tribromide perovskites”, *Sci. Adv*., 2020, **6**, eabb6393.
23. A. Musiienko, J. Pipek, P. Praus, *et al*., “Deciphering the effect of traps on the electronic charge transport properties of methylammonium lead tribromide perovskites”, *Sci. Adv*., 2020, **6**, doi: 10.1126/sciadv. abb6393.
